# Effect of *Artemisia dracunculus* L. Water Extracts on *Aphis fabae* Scop. and Its Predators in Field Conditions

**DOI:** 10.3390/molecules31193399

**Published:** 2026-09-24

**Authors:** Janina Gospodarek, Natalia Kwiecień, Agnieszka Krajewska, Dorota Mańkowska, Iwona B. Paśmionka

**Affiliations:** 1 Department of Microbiology and Biomonitoring, University of Agriculture, Al. A. Mickiewicza 21, 31-120 Krakow, Poland; natalia66@autograf.pl (N.K.); iwona.pasmionka@urk.edu.pl (I.B.P.); 2 Institute of Natural Products and Cosmetics, Faculty of Biotechnology and Food Sciences, Lodz University of Technology, Stefanowskiego 2/22, 90-530 Lodz, Poland; agnieszka.krajewska@p.lodz.pl (A.K.); dorota.mankowska@p.lodz.pl (D.M.)

**Keywords:** black bean aphid, tarragon, water extract, natural enemies, antioxidant activity, volatile compounds

## Abstract

Use of plant-based aqueous extracts offers a potentially simple and low-cost approach to pest control. However, their effectiveness under field conditions remains poorly recognised. The aim of the study was to evaluate the effectiveness of aqueous extracts prepared from dried and fresh *Artemisia dracunculus* L. plant material (D-Es and F-Es, respectively) in controlling *Aphis fabae* Scop. in field conditions. D-Es were applied in concentrations of 2% (D2), 5% (D5) and 10% (D10) and F-Es in concentrations of 10% (F10), 20% (F20) and 30% (F30). A reduction in the *A. fabae* population was observed with the use of both D-Es and F-Es (although not as strong as with chemical control (Ch)), with the greatest effect observed after using D-E at a concentration of 10% (D10-E). In the case of D-Es, the aphid-suppressing effect increased as the extract concentration rose. No similar result was observed for F-Es. Regardless of the concentration used, the individual F-Es did not differ significantly from one another. The strength of the limiting effect on *A. fabae* was arranged in the following order of treatments: Ch > D10 > F10 > D5 > F20 > D2 > F30. Analysis of the volatile compounds contained in D10-E revealed that the main constituents were methyleugenol (27.7%), elemicine (15.6%), (*E*)-isoelemicine (12.5%), terpinen-4-ol (9.3%) and linalool (4.3%). The extract exhibited the highest antioxidant activity and the highest concentration of polyphenolic compounds. Reduced numbers of the main aphid predators (syrphids and ladybirds) in the treatments with extracts went along with lower prey availability; however, the predator–prey ratio in these treatments was more favourable than in the control. This suggests that the use of F-Es and D-Es does not adversely affect predator abundance. The ease of preparation and availability of the material make D10-E suitable for use on organic farms or as part of integrated pest management.

## 1. Introduction

Plant-based extracts used to control herbivores have recently been the subject of numerous studies, given the transition from chemical to green management [1]. The mechanism by which botanical extracts act against pests is linked to the presence of bioactive secondary metabolites, which may deter pests from colonising plants (by affecting olfactory stimuli) or exert an insecticidal effect. The fact that the extracts are mixtures of many (often several dozen) components, whose mechanisms of action against pests may vary, suggests that the risk of pests developing resistance will be low [2,3]. The use of botanical extracts prepared using cold water as insecticides is a beneficial solution due to the ease of their preparation (no special equipment is required), low costs (no need for expensive solvents or energy to heat water), and the possibility of preparing the extract oneself directly on farm, using plant material available on site and at the time the treatment is required [4]. Furthermore, they are regarded as environmentally friendly and readily biodegradable [5]. The main limitations, however, are as follows: inconsistent practical efficacy resulting from fluctuations in environmental conditions during cultivation, low stability, and low persistence [2].

Tarragon (*Artemisia dracunculus* L.) is a herbaceous, perennial plant native to Eastern Europe, parts of Asia and North America, characterised by vigorous growth, which is an advantage in terms of the ease of obtaining material for the preparation of extracts. It is used both as a spice and for its medicinal properties [6,7]. Previous laboratory studies have focused on the use of essential oils from this plant to control, amongst others, storage pests [8,9,10], aphids [11,12], thrips [11], whiteflies [13], the Colorado potato beetle (*Leptinotarsa decemlineata* Say) [14] and sanitary pests [15,16,17]. However, there is considerably less information available on the use of aqueous extracts from this plant against pests. Most of the existing research has been conducted under laboratory conditions, while field-based evidence remains limited. To date, laboratory studies have tested the effectiveness of aqueous extracts from dried tarragon herb (D-Es) and from fresh plant material (F-Es) in controlling the pea aphid (*Acyrthosiphon pisum* Harris), Colorado potato beetles, cereal leaf beetles (*Oulema melanopa* L.) [18], pea leaf weevils (*Sitona lineatus* L.), and black bean aphid (*Aphis fabae* Scop.) [19,20]. In this context, significant aphicidal properties were observed for F-E at a concentration of 30% and D-E at a concentration of 10% against nymphs and wingless females of *A. fabae* [20], whilst these extracts had no adverse effect on the voraciousness of ladybird *Coccinella septempunctata* L. L3 larvae. The above laboratory test results, therefore, provide a basis for verifying the effectiveness of water extracts of tarragon against aphids under field conditions. The available literature lacks information on the use of tarragon aqueous extracts in the field. In particular, studies that also take into account the impact on non-target organisms, i.e., natural enemies of pests, are rare.

The black bean aphid (BBA) is a polyphagous species of great economic importance, attacking both cultivated and wild plants. Its feeding reduces both the quantity and quality of the crop. It is also a vector of dangerous viral diseases [21,22]. Under natural conditions, key factors limiting its population include beetles of the ladybird family (Coleoptera, Coccinellidae), flies of the hoverfly family (Diptera, Syrphidae), lacewings (Chrysopidae, Neuroptera), anthocorids (Anthocoridae, Heteroptera), spiders (Arachnida, Araneae) and other polyphagous predators [23]. Chemical treatments are currently the primary method of controlling BBA. These often need to be repeated several times. This can result in pests developing resistance to the active ingredients in insecticides, as well as having adverse effects on beneficial fauna [21,23].

The aim of the study was as follows: (i) to evaluate the effectiveness of aqueous extracts from dried and fresh tarragon in controlling BBA in field conditions and (ii) to assess the effect of these extracts on aphid predators. An analysis of volatile compounds from fresh (FH) and dried (DH) *A. dracunculus* herb and water extracts was carried out, and the total polyphenol content and antioxidant activity of the extracts were measured.

## 2. Results

The extracts used in the experiment were prepared at concentrations conventionally defined as 2%, 5% and 10% for D-E (dried plant material [g] + cold redistilled water [mL] in ratios of 2:100 (D2-E), 5:100 (D5-E) and 10:100 (D10-E)) and 10%, 20% and 30% for F-E (fresh plant material [g] + cold redistilled water [mL] in ratios of 10:100 (F10-E), 20:100 (F20-E) and 30:100 (F30-E). After 24 h exposure, the individual mixtures yielded the following volumes of ready-to-use extracts [mL]: D2-E—84, D5-E—83, D10-E—71, F10-E—88, F20-E—85, F30-E—84.

### 2.1. Composition of Volatile Compounds

The qualitative and quantitative compositions of volatile compounds in *A. dracunculus* L. fresh and dried herb and water extracts were investigated. The results are shown in Table 1 and Figure 1. The compounds are listed in the order they leave the chromatography column.

Volatile compounds from FH and DH were isolated and analyzed using the HS-SPME-GC-FID-MS method. The monoterpene hydrocarbons and phenylpropanoids were the major groups of volatile constituents regardless of the type of plant material. Sabinene (9.4% in FH, 18.8% in DH), (*E*)-β-ocimene (7.6% in FH, 6.7% in DH), γ-terpinene (5.9% in FH, 3.1% in DH) were the major constituents among monoterpene hydrocarbons. Methyleugenol (17.6% in FH, 16.6% in DH) and elemicin (3.3% in FH, 4.0% in DH) were dominant among phenylpropanoids. Comparing the qualitative and quantitative composition of volatile compounds in both plant materials, some differences can be observed. A higher content of monoterpene hydrocarbons and a lower content of phenolic compounds were observed in FH. However, the ratio of sesquiterpene hydrocarbons was higher in DH. β-Bourbonene, α- and β-isocomene, β-cubebene, α-gurjunene, β-caryophyllene, β-copaene, aromadendrene, cadina-3-,5-diene, (*E*)-β-farnesene, α-humulene, ε-, γ- and α-muurolene, germacrene D, ε-, α- and δ-cadinene were found among the constituents; however, their content does not exceed 1%.

In all water extracts, regardless of type of plant material used (fresh, dried), a decrease in the content of non-polar compounds, i.e., hydrocarbons, was observed, compared to their content in plant material. The content of monoterpene hydrocarbons was from 5.6 to 6.6% and 8.5 to 12.1% in F-Es and D-Es, respectively. The total content of sesquiterpene hydrocarbons was less than 1% in both cases. The major group of constituents in water plant extracts of *A. dracunculus* was phenylpropanoids. The total content of these constituents ranged from 69.5 to 76.5% for F-Es and from 60.6 to 71.0% for D-Es, which was approximately three-times higher than in the plant material. The second-largest group was oxygenated monoterpenes. The total content of these constituents ranged from 15.4 to 23.1% and 16.3 to 23.1% in F-Es and D-Es, respectively. Methyleugenol (38.2–55.7% for F-Es, 27.7–39.4% for D-Es) and elemicin (11.7–24.2% for F-Es, 12.7–15.6% for D-Es) were detected as major constituents of water extracts. It was observed that the content of methyleugenol decreases while the content of elemicin increases with increasing concentration of the F-Es. In the case of D-Es, however, a decrease in the methyleugenol content and an increase in the elemicin content were observed at the highest concentration, i.e., 10%, compared with the 2% concentration. In the D-Es, an increased content of (*E*)-isoelemicin (12.5–15.8%) was also observed, while in the F-Es, it did not exceed 4%. The content of this constituent decreased as the concentration of the active substance in the F-Es increased, whilst in the case of D-Es, it increased at a concentration of 5% and then decreased at a concentration of 10%. The differences in the quantitative and qualitative composition between the D-Es and F-Es are comparable to the differences observed in the case of corresponding plant material: in the DH, a higher content of (*E*)-isoelemicin and a lower content of methyleugenol were observed compared to the FH.

### 2.2. Antioxidant Activity and Total Content of Polyphenols

The highest antioxidant activity was characterised by the D10-E (9.045 mM TEAC (Trolox equivalent antioxidant capacity)) and the lowest by the F10-E (1.673 mM TEAC) (Table 2). The antioxidant activity of all tested extracts is arranged in order: D10-E > D5-E > F30-E > F20-E > D2-E > F10-E. The free radical scavenging capacity (RSC) of the tested extracts ranged from 17.49% (F10-E) to 87.99% (D10-E). In turn, the EC_50_ value was 3.52% and 25.62% for the extracts obtained from DH and FH, respectively (Table 2, Figure 2). The ANOVA revealed statistically significant differences between the study groups (F(5.12) = 231,456.93; *p* < 0.001).

The highest TCP (total content of polyphenols) for F-Es was detected at the 30% concentration (0.814 mg GAE/g) and at 10% concentration for D-Es (0.899 mg GAE/g) (Table 2).

### 2.3. Effect of Extracts on BBA

Statistical analysis revealed a significant effect of treatments, the year of the study, and the interaction between these two factors on the occurrence of BBA (Table S1). Analysis carried out separately for each year also showed a significant effect of treatments. During the 2023 season, aphids were significantly less numerous than in the other two seasons (Table S2). Chemical control was the most effective method for reducing aphid populations (Figure 3, Table S2). Among the tarragon water extracts tested, D10-E proved to be the most effective. In the case of D-Es, the aphid-suppressing effect increased as the extract concentration rose. No similar effect was observed for F-Es. Regardless of the concentration used, the individual F-Es did not differ significantly from one another in any of the years of observations. It is worth noting that in the 2023 season, the efficacy of both D-Es and F-Es (except for F30-E) was at a similar level to that of chemical control (Ch). An analysis carried out independently of the year of the study confirmed a significant limiting effect of both D-Es and F-Es on the occurrence of BBA, with this effect ranked in the following order across the individual treatments: Ch > D10 > F10 > D5 > F20 > D2 > F30 (Table S2). An analysis of the interaction between treatment and year confirmed the trends identified by analysing the data from individual seasons separately, whilst also showing that, in the 2024 season, D10-E was placed in the same homogeneous group as Ch.

The average results for individual seasons presented above are reflected in the course of aphid population dynamics (Figure 4, Table S3). In each year, a significant effect of treatment and observation date was observed, whilst in the 2024 season, an interaction between these two factors was also significant. Significant differences between treatments generally became apparent during the initial stage of aphid population development, and in 2022 and 2024, also during the final stage. Despite the application of treatments using both chemical preparations and extracts, a gradual increase in aphid abundance was observed, which generally peaked at the turn of May and June.

### 2.4. Effect of Extracts on Aphid Predators

Syrphidae density was strongly related to aphid abundance (Figure 5, r = 0.48). As with BBA, both treatment and year had a significant effect on their abundance, and the interaction between these two factors was also significant (Table S1). These predators were most abundant in the 2024 season, followed by 2022, and least abundant in 2023, which is consistent with the data on aphid occurrence (Table S2). Analysis of each year separately revealed a significant effect of treatments. In 2022, Syrphidae were most abundant in the control plot. The use of all types of F-Es, as well as D5-E and D10-E, had a suppressing effect on the abundance of these predators. In contrast, during the 2023 season, such a limiting effect compared with the control was observed only for D5-E and D10-E. By contrast, during the 2024 season, no significant effect of either F-Es or D-Es on the occurrence of these predators was recorded. An analysis of the effect of the treatments, regardless of the year of the study, showed that, apart from the Ch treatment, the Syrphidae population was lower than in the control group only in D5 and D10 (Table S2). The analysis of the interaction between treatments and year was mostly consistent with the results of the analyses for individual years taken separately; however, it did not reveal any negative effect of D5-E and D10-E in the 2023 season (the results for these treatments were in the same homogeneous group as the control).

An analysis of Syrphidae abundance at specific observation dates revealed a significant effect of both the observation date and the treatment on this parameter (Figure 6, Table S3). In the 2022 season, Syrphidae were most abundant at the end of May, and significant differences between treatments were also recorded at that time. In the 2023 season, the abundance of Syrphidae was significantly lower than in other seasons, and no statistically significant differences were found in the individual observation dates, whereas in the 2024 season, hoverflies reached their peak abundance around mid-June. In that season, the only statistically significant difference across the various observation dates was recorded on 5 June and concerned a significantly lower abundance of Syrphidae in the chemically protected site compared with the other sites.

Another frequently observed group of predators was ladybirds. Statistical analysis revealed a significant effect of the year of the study, the treatments and the interaction between these factors (Table S1, Figure 7). Ladybirds were recorded in the greatest numbers in the 2022 season, followed by 2023, and in the smallest numbers in the 2024 season (Table S2). In the analysis carried out for each year separately, no significant effect of the treatments under study on the occurrence of ladybirds was found in the 2023 season. In the 2022 season, ladybirds were most abundant in the control. Their abundance in the treatments where both F-Es and D-Es were used was significantly lower, though higher than in the plot treated with chemical preparations (with the exception of D10). In contrast, ladybird populations were at their lowest in the 2024 season. None of the treatments tested using F-Es and D-Es differed from the control in this respect. However, significantly higher ladybird populations were recorded in the treatments using F-Es than in those using synthetic preparations (Ch). Analysis of the data, regardless of the year of the study, showed a lower number of ladybirds in treatments Ch, D5, D10, F10 and F20 compared with the control (Table S2). An analysis of the treatment and year interaction revealed a higher abundance of ladybirds in D10 than in Ch during the 2023 season, whilst in the 2024 season, it did not confirm any differences between Ch and F-Es.

An analysis of the dynamics of Coccinellidae occurrence revealed a significant influence of both treatments and the date of observation (Figure 8, Table S3). The highest abundance of ladybirds was recorded in the 2022 season, with peak numbers occurring between 5 and 15 June, which coincides with the previously noted decline in aphid numbers (Figure 4). In the 2023 and 2024 seasons, ladybirds were recorded less frequently, and their peak occurrence (10–13 June 2023 and 12–16 June 2024, respectively) also coincided with a decline in aphid numbers (Figure 4).

Other predators in general also differed significantly depending on the year of the study and the treatments; a significant interaction between these two factors was also observed (Table S1). In the 2023 season, significantly fewer other predators were recorded than in the 2022 and 2024 seasons (Table S2). Analysis of individual years revealed a significant effect of treatment only in the 2024 season (Figure 9, Table S1). This group of predators was recorded in the highest numbers in treatment D5, followed by treatment F30. These treatments differed significantly from the control. The lowest numbers of other predators were recorded on chemically protected plants. Analysis of the data, regardless of the year, showed a significantly higher abundance of this group in D5 and F20 than in the control (Table S2). In turn, analysis of the interaction between treatments and year showed a significantly higher abundance of other predators in F20 compared with the control in the 2022 season, whilst in the 2024 season, higher numbers were recorded in D5 and F30 than in the control.

An analysis of the dynamics of the occurrence of other predators in the 2022 season revealed a significant effect only of the observation date (Table S3), whilst in the 2024 season, both the observation date and the treatment had a significant effect; however, no interaction between the two factors was found. In contrast, an analysis conducted separately for each observation date revealed no statistically significant differences. The dynamics of the occurrence of other predators in the 2022 and 2023 seasons were subject to considerable fluctuations (Figure 10). In contrast, in the 2024 season, this group was most abundant, with peak numbers occurring between 21 May and 3 June, which coincided with the peak occurrence of aphids.

Since the presence of predators under field conditions is inextricably linked to the availability of food (aphids) and it is not possible to separate the effects of the treatments on these two components, two indices were used, which integrate the treatments, aphid abundance and predator abundance: namely, the predator–prey ratio for the main predators, i.e., Syrphidae larvae and Coccinellidae larvae and adults, and the Pearson correlation coefficient between aphid and predator abundance. PCA analysis was also performed for aphids and predators.

The number of aphids per one predator from the Syrphidae family was significantly influenced by both the treatments and the year of the study (Table S4). Over the course of three seasons, the prey–predator (Syrphidae larva) ratio was least favourable in the control (Table 3). In the 2022 season, there were significantly fewer aphids per one Syrphidae larva in treatments D10, F10 and F20 compared with the control, whereas in the 2024 season, a similar trend was observed in all protected treatments compared with the control. In the 2023 season, no statistically significant differences were observed in this respect.

The number of aphids per single Coccinellidae larva was significantly influenced by the year of the study (Table S4). The values for this parameter were generally lower than those for syrphids, and in the 2022 and 2023 seasons (when the differences were statistically significant), they were least favourable in the control (Table 3). In all other treatments, there were significantly fewer aphids per predator than in the control. The most favourable predator–prey ratios were recorded in D10 and D5 in the 2022 and 2023 seasons, respectively. In the 2022 season, this parameter also reached more favourable (lower) values in the F-E- and D-E-protected plots than on chemically protected plants; in the 2023 season, this trend persisted for D5 and D10.

The number of aphids per one adult ladybird was influenced by the year of the study (Table S4). An analysis carried out for each year separately revealed a significant effect of treatments only in the 2022 season (Table S4 and Table 3). More favourable predator–prey ratios were recorded at all protected plots than at the control. Among the treatments using F-Es and D-Es, the ranking of this parameter was as follows: D10 < D5 < F20 < F10 < F30 < D2. In the 2023 and 2024 seasons, no significant effect was observed.

Of the three groups of predators, the Syrphidae showed the strongest correlation with aphids (Table 4). The lowest correlation coefficient was recorded in the Ch treatment, whilst in some treatments where extracts were used (D2, D10, F10, F30), these values were higher than in the control. In the case of Coccinelidae, the highest correlation with aphids was found in the control, followed by D5; in the remaining treatments, this coefficient was not statistically significant. In contrast, for other predators, a positive, statistically significant correlation with aphids was recorded in D5 and F10.

PCA analysis of the occurrence of aphids and predators on broad bean plants treated with tarragon extracts at various concentrations showed that the first and second ordination axes together explain 26.32% of the variation (Figure 11). Aphids and predators were clearly positively correlated with the first axis, whilst a clear positive correlation with the second axis was found for other predators and, to a lesser extent, for Coccinellidae. The analysis also confirms a clear negative effect of Ch treatments on aphids.

The broad bean seed yield was influenced by both the treatments and the year of the trial. The interaction between these two factors was also significant (Table S5). The highest yield was recorded in the 2024 growing season, followed by 2023, whilst the lowest was recorded in 2022 (Table S6). Analysis of the data from each season separately also revealed a significant effect of the treatments (Table S5, Figure 12). Among the treatments tested using F-Es and D-Es, the highest seed yield was recorded in D10 in two out of three seasons (2022 and 2024). This yield was on a similar level to that obtained in the Ch treatment. In all three seasons, the use of almost all F-Es and D-Es resulted in a significant increase in yield compared with the control. The exception was the 2022 season, in which treatments D2 and D5 did not differ significantly from the control.

An analysis of the effect of treatments, regardless of the year, showed that the yield in treatment D10 was on a similar level to that in Ch, and also confirmed the beneficial effect of all protective measures on this trait (Table S6). An analysis of the interaction between treatment and year revealed relationships consistent with those found in the analysis carried out for each year separately.

## 3. Discussion

### 3.1. Composition of Volatile Compounds

The chemical composition of the essential oil of *A. dracunculus* is fairly well documented. Phenylpropanoids predominate (with estragole, elemicin, eugenol and their derivatives being the most common). The most frequently identified monoterpenoids are sabinene, terpinen-4-ol, β-ocimene, *cis*-ocimene, α-trans-ocimene and limonene [24,25]. The composition of volatile compounds in the plant material and in the D-Es and F-Es used in this experiment did not differ substantially from reports in the literature concerning essential oils from this plant. Methyleugenol and elemicin were identified as the major constituents. Terpinen-4-ol, listed as a component of tarragon essential oil (up to 41%) [25], reached a concentration of up to 9.3% in aqueous extracts in this experiment. The available literature lacks information on the composition of water extracts from *A. dracunculus.* Studies into the potential toxicity of estragole and methyleugenol to human health, using, amongst other, an aqueous extract of tarragon, indicate only that extracts prepared using hot water contain very low concentrations of both these compounds compared with ethanol extracts [26].

### 3.2. Antioxidant Activity and Total Content of Polyphenols

The antioxidant activity of tarragon extracts was higher in extracts prepared from dried rather than fresh plant parts. This was reflected in the RSC and in the TCP values. The antioxidant potential of *A. dracunculus* is directly linked to its polyphenol content [27,28]. Data on the TCP content in tarragon extracts or essential oil vary depending on the processing methods used (drying, freezing) and the type of solvent or plant part employed, ranging from 0.451 mg GAE/g of sample for essential oil [29], 32.91–42.53 g GAE/kg of extract for freeze-dried water–ethanol extracts [30], 192.1 mg GAE/g dry weight for methanol extracts to 168.3 mg GAE/g dry weight for the aqueous extract [28]. In turn, EC_50_ values ranged from 17.42–24.27 µg/mL for extracts prepared using hot water [27] to 231 µg/mL for a hydroethanolic extract [31]. In light of the results cited above, it can be concluded that the studied extracts of *A. dracunculus* (with TCP content of 0.528–0.899 mg GAE/g of extract, and EC_50_ = 3.523% and 25.616% for D-Es and F-Es, respectively) were a good source of compounds with high antioxidant capacity.

### 3.3. Effect of Extracts on Aphis fabae

The studies showed a reduction in BBA populations when using both D-Es and F-Es, with the strongest effect observed at the highest D-E concentration—10%. Depending on the year of the study, the average number of aphids per plant in this treatment was approximately 5- to 18-times lower than in the control. There are no data in the available literature on the efficacy of aqueous tarragon extracts against aphids under field conditions. Under laboratory conditions, D10-E caused a 78% mortality of nymphs and wingless females of BBA after 108 h [20]. The effect of F-Es in those studies was slightly weaker, which is consistent with the results of the present experiment. Aqueous extracts from plants of the Asteraceae family (*Ageratum conyzoides* (L.), *Chromolaena odorata* (L.), *Synedrella nodiflora* (L., Gaertn.)), of which *A. dracunculus* is a representative, demonstrated efficacy in controlling the cabbage aphid *Brevicorynae brassicae* (L.) similar to that achieved with the synthetic pesticide Attack^®^ (emamectin benzoate) [32].

The observed effect of tarragon extracts on BBA may have been due to the presence of compounds with insecticidal or repellent properties. All the extracts used in this study were characterised by a high content of phenylpropanoids (60.6–76.5%). These substances exhibit significant insecticidal activity [33,34]. The mechanism of insecticidal action of phenylpropanoids is most likely based on their effect on the respiratory system. They proved to be weak AChE (acetylcholinesterase) inhibitors but caused a significant increase in cAMP levels, suggesting an effect via interference with the octopaminergic system [33].

What distinguished D10-E (the most effective extract against aphids) from the other F-Es and D-Es was its highest content of monoterpene hydrocarbons (12.1%), a fairly high content of oxygenated monoterpenes (23.1%), and the lowest content of phenylpropanoids (60.6%).

Among the monoterpene hydrocarbons, D10-E had the highest content of compounds: myrcene (0.4%), α-terpinene (1.9%), *p*-cymene (1.3%), β-phelleandrene (1.0%), γ-terpinene (3.6%), *p*-cymenene (0.3%) and terpinolene (0.9%). Monoterpene hydrocarbons are known for their insecticidal properties against aphids [35,36,37], although the available data relate to essential oils and typically higher concentrations than those found in this experiment. β-myrcene (2.9%) was one of the main constituents of *Pinus peuce* (Griseb.) essential oil, which, within 24 h of application, demonstrated 100% efficacy against the aphid species *Sitobion avenae* (Fabr.) and *Rhopalosiphum padi* (L.) [35]. It was also detected in *Thuja occidentalis* (L.) essential oil (at a concentration of 14.62%), which exhibited 100% mortality of the aphid *Myzus persicae* (Sulzer) 18 h after application at a concentration of 5.0 µL/L [36]. *P*-cymene was present in the essential oil of *Origanum vulgare* subsp. *hirtum* (Link) at a concentration of 13.7%. This essential oil vapours demonstrated 100% efficacy against *M. persicae* at doses of 2 μL/L air and 3 μL/L air [37].

Of the oxygenated monoterpenes, D10-E had the highest terpinen-4-ol content (9.3%) among the extracts studied. Terpinen-4-ol has been identified as the main constituent of *Melaleuca alternifolia* (Maiden & Betche) essential oil. This essential oil exhibited significant insecticidal activity and a deterrent effect against the aphid *M. persicae* [38]. Under the influence of this oil, enhanced upregulation of the *OSD*, *TOL*, *ANT*, *HSP60* and *FPPSI* genes, responsible for the negative impact on the fertility, growth and development of *M. persicae*, was identified. Terpinen-4-ol also exhibited significant insecticidal activity against *Sitophilus zeamais* (Motschulsky). Following fumigation with this compound, the expression levels of detoxification genes were altered, particularly for P450s [39]. Moreover, terpinen-4-ol induced marked repellent behaviour in the stable fly *Stomoxya calcitrans* (L.) [40]. On the other hand, it is cited as an attractant for *Ceratitis capitata* (Wiedemann) [41].

D10-E was also characterised by the highest content of the oxygenated monoterpene linalool (4.3%) amongst the extracts studied. The role of this compound in insect behaviour is diverse. Studies on the aphicidal activity of *Laurus nobilis* (L.) essential oil, one of the main identified constituents of which was linalool (10.19%), demonstrated its high efficacy against BBA under field conditions (an 86.5% reduction at a 20% EC concentration) [42]. However, in studies on the use of plant-derived substances against third-instar *B. procera* larvae, linalool showed no toxicity [33]. The addition of linalool to ginger root oil resulted in the capture of more male *C. capitata* than ginger root alone [41]. Linalool was also one of the compounds identified in the headspace of *Vicia faba* (L.) plants in studies investigating the volatile compounds responsible for the BBA locating its host plant; it is listed as an alarm pheromone inhibitor [43].

Methyleugenol (phenylpropanoid), when tested as a single compound at a 13.55% concentration, did not exhibit aphicidal activity as strong as that of the main component of *L. nobilis* essential oil—1,8-cineole (26.43%) [42]. In the present study, methyleugenol was present at a concentration of 27.7% in the D10-E and was the main component of the extract; however, its concentration was lower than in the D5- and D2-Es and considerably lower than in the less effective F-Es.

In summary, the response of aphids may vary when they are exposed to a mixture of volatile compounds, as compared to their reaction to individual ingredients. Moreover, BBA is a species that feeds on a large number of plants, and it locates these plants by recognising species-specific ratios of commonly occurring plant volatiles [43]. Thus, the specific mixture of volatile compounds supplied with D10-E may have made the broad bean less attractive to the BBA.

D10-E was characterised by the highest antioxidant activity. Similarly, in a study by Nawarathne et al. [44], the water/acetone leaf extract of *Psophocarpus tetragonolobus* (L.), which exhibited the highest activity against BBA, showed the highest antioxidant activity among the different types of extracts from this plant (water/acetone, cold water, hot water). The authors attributed this effect to the highest concentration of polyphenolic compounds. In the present experiment, the D10-E also exhibited the highest TPC content. An increased content of natural antioxidants such as polyphenols is a common response to stress factors such as drought, pollution, feeding by pests (including aphids) [45]. The presence of higher concentrations of TPC in the D10-E might, therefore, discourage the pest from feeding.

### 3.4. Effect of Extracts on Aphid Predators

The density of hoverflies varied in different years of research, but they were generally most abundant in the control. Their abundance on plants where F-Es and D-Es were applied in 2022 and 2023 was lower than in the control plots, with D-Es having a greater limiting effect on Syrphidae abundance. On plants where chemical control was applied, the presence of Syrphidae was negligible. The extracts tested may have affected the hoverflies directly but also indirectly by reducing their food source—aphids. Under field conditions, where both components (predators and aphids) are present simultaneously and are closely interlinked, it is, therefore, not possible to draw any definitive conclusions regarding the causes of the observed effects. An indicator that, to some extent, allows for the integration of data on the impact of treatments on aphid and predator populations is the predator–prey ratio. This is also a good indicator of the potential effectiveness of predators in controlling aphids [46,47]. The number of aphids per Syrphidae larva was significantly lower at sites where F-Es and D-Es were applied compared with the control (1.64–4.31 times in the 2022 season; 3.41–6.53 in the 2023 season; 3.50–7.11 in the 2024 season, depending on the treatment). The most frequently recorded species of Syrphidae in this experiment was *Episyrphus balteatus* (Deg.). According to Wnuk [46], a single larva of this species can completely destroy a BBA colony at a predator–prey ratio of 1:15–1:50 within 1.5–4 days, and at a ratio of 1:200 within 7–8 days. At a ratio of 1:300, the larva significantly reduces the BBA colony growth rate. The predator–prey ratio values calculated in this experiment indicate that, in the 2023 season, Syrphidae larvae were able to effectively control the BBA population at all sites except the control. Furthermore, in some treatments during other seasons, the predator–prey ratios approached the 1:300 level (D10 and F10 in 2024). This suggests that the use of F-Es and D-Es does not adversely affect the occurrence of syrphid flies. Further evidence that the natural co-occurrence of aphids and syrphids was not disrupted in treatments using extracts is provided by the high correlation coefficients between these two components of the community, which in some cases are even higher than in the control. Female hoverflies, when searching for food for their future offspring, are guided primarily by olfactory and visual cues from aphids (the smell of aphids, the presence of aphid alarm pheromone, the scent of honeydew, and the shape and movement of aphids), whilst the decision to lay eggs is made following an additional visual and tactile assessment during which they evaluate the size of the colony and, thus, its suitability as a food source capable of meeting the nutritional requirements of newly hatched larvae [48]. Therefore, the lower abundance of Syrphidae in the areas treated with extracts was most likely a consequence of the reduced availability of prey.

The available literature lacks data on the occurrence of hoverflies under field conditions when aqueous extracts of *A. dranunculus* are used. The occurrence of hoverflies (with *E. balteatus* as the dominant species) in treatments using aqueous extracts from other plant species of the Asteraceae family (*A. conyzoides, C. odorata, S. nodiflora*) did not differ significantly from the water control but was significantly higher than in treatments using the synthetic pesticide Attack (which is similar to the results of this experiment) [32]. There are also few data available on the effect of the compounds present in F-Es and D-Es on hoverflies. A binary combination of methyl salicylate and linalool in an apple orchard attracted Syrphidae, with the effect being stronger than when each component was applied separately [49]. In the present study, the presence of both these components was recorded in the tested extracts. Studies on the olfactory responses of *E. balteatus* to plant volatiles have shown that monoterpenes induced responses, whilst there was no response to sesquiterpenes, with the exception of (*E*)-β-farnesene, which is an aphid alarm pheromone. The latter compound was detected in trace amounts only in FH, whilst it was not found in F-Es or D-Es. The authors emphasise that predatory hoverfly oviposition is determined by a mixture rather than by a single chemical signal [50].

Two species of ladybird, *Coccinella septempunctata* (L.) and *Harmonia axyridis* (Pallas), were predominant in the present experiment. The occurrence of ladybirds in BBA colonies was correlated with aphid abundance, i.e., with the highest aphid density in the control and the lowest in the chemical treatment (r = 0.20). However, the correlation coefficients for the individual treatments did not reach such high values as those observed for Syrphidae. Analysis of the data, regardless of the year of the study, showed a lower number of ladybirds in treatments Ch, D5, D10, F10 and F20 compared with the control. However, this parameter was significantly higher in all treatments using extracts than in Ch. Analysis of the dynamics of predator occurrence indicates that their numbers peaked after the period of maximum aphid abundance in individual plots, suggesting that the availability of food—aphids—plays a significant role as the primary factor determining predator occurrence (similarly as in the case of hoverflies). The predator-to-prey ratio for ladybird larvae in two of the three study seasons was significantly lower in all treatments where extracts were applied than in the control, indicating that the extracts did not have a negative impact on the abundance of these predators. A similar effect was also observed in one study season for adult Coccinellidae. Ladybirds are capable of effectively limiting aphid populations at a predator–prey ratio of 1:150 [47]. In the 2022 and 2023 seasons, in treatments with F-Es and D-Es used, the predator–prey ratios calculated for ladybird larvae ranged from 1:29 to 1:78, indicating the potential for effective aphid control.

The available literature lacks data on the effect of aqueous tarragon extracts on ladybirds under field conditions. In laboratory experiments, no significant differences were observed in the number of aphids consumed by L3 larvae of *C. septempunctata* when tarragon water extracts were used [20], which also indicates that F-Es and D-Es have no negative impact on these predators. *Coccinella magnifica* (Redtenbacher) was recorded in lower numbers in plots treated with water extracts from plants of the Asteraceae family than in the water control but significantly more numerous than in the treatment using the synthetic pesticide Attack, which is consistent with the trends observed in this experiment [32].

Some volatile compounds, including those found in the extracts studied, are known for their ability to attract ladybirds. Predator–prey ratios in the plots where the most effective extract—D10-E—was applied were among the most favourable of all the extract types and concentrations tested, reaching their lowest values in the 2022 and 2024 seasons for Coccinellidae larvae and in the 2022 and 2023 seasons for adult Coccinellidae. Terpinen-4-ol, which was present at the highest concentration in D10-E, is cited in the literature as a compound that acts as an attractant for adult ladybirds of the species *H. axyridis* [51]. Similarly, linalool is listed as a substance highly attractive to *H. axyridis* [52] and *Propylaea japonica* (Thunberg) [53]. Two other volatile sesquiterpenes, (*E*)-β-farnesene and (−)-β-caryophyllene, listed as attractants for *H. axyridis* [50] were not detected in the extracts tested, whilst their presence in the plant material was negligible. Among the analysed components in the tested extracts, an increased content of eugenol was also noted compared to its trace levels in the plant material, with extracts based on fresh material exhibiting higher concentrations. Of the 58 terpenoids, aromatic, aliphatic and other structural compounds from flowers that were evaluated via behavioural responses in ladybird tube olfactometer assays, eugenol was significantly attractive to *C. septempunctata* [52]. Methyl salicylate, identified as an attractant for both *H. axyridis* and *C. septempunctata* in the extracts studied, was also present, albeit in small quantities. In studies testing the attractiveness of mixtures of selected compounds to enhance the attraction and luring of ladybirds, the best results were obtained using a mixture of α-humulene:β-pinene/methyl salicylate/indole = 1:3:3:10. Of the compounds listed, β-pinene and α-humulene were not detected in the extracts tested, although they were present in the plant material [52]. A mixture of farnesene and linalool used in traps proved to be an attractant for Coccinellidae [49]. Beta-myrcene (present in the highest concentration in fresh tarragon herb—4.8%, but in small amounts in F-Es and D-Es: 0.2–0.4%) was identified as a component that increased the attraction of *H. axyridis* to *Cnidium monnieri* (L.) Cusson plants infested by aphid *Semiaphis heraclei* (Takahashi) [54].

Among other predators present in the BBA colonies were lacewings, anthocorids and spiders. In two of the three study seasons, there were no differences in their occurrence between the individual treatments, whilst in the third season, in the F-E- and D-E-protected plots—with the exception of D2—they were more abundant than in the control (differences significant for D5 and F30). They were found in the lowest numbers at the chemically protected site. The available literature provides some information on the effects of the compounds present in the extracts under investigation on lacewings. Research into the use of volatile compounds to attract predators has shown that a mixture of methyl salicylate and farnesene as well as linalool is highly attractive to *Chrysoperla carnea* (Stephens) sensu lato [49]. Methyl salicylate was present in the extracts studied in very low quantities, whilst farnesene was not present; linalool, on the other hand, occurred in higher concentrations in D-Es than in F-Es. Linalool is also highly attractive to *Chrysopa phyllochroma* (Wesmael), significantly enhancing female oviposition [55]. A binary mixture of beta-citronellol and linalool was recommended as an efficient substance for the biological control of the aphid *Metopolophium dirhodum* (Walker) due to its non-toxicity to the larvae of *Aphidoletes aphidimyza* (Rond.) and *C. carnea* [56]. Among other compounds present in the F-Es and D-Es, lacewings were also attracted to eugenol [57]. Spiders constituted a significant group amongst other predators. In the study by Amaobeng et al. [32], they were more abundant in treatments using botanical extracts from plants of the Asteraceae family than in those where the synthetic pesticide Attack was used, which is consistent with the present results.

In two out of the three years of the study, the highest broad bean seed yield was obtained in the treatment that was most effective at controlling BBA, i.e., D10, and this yield was at a similar level to that achieved with chemical treatments. BBA is the main factor limiting broad bean growth and, if control measures are not taken, can lead to a three- to four-fold reduction in yield [23].

In summary, chemical control was the most effective method for reducing aphid populations. In the case of extracts based on dried material, the efficacy increased with higher concentrations, with the 10% concentration proving to be the most effective. Analysis of the volatile compounds in this extract revealed methyleugenol (27.7%), elemicin (15.6%), (*E*)-isoelemicin (12.5%), terpinen-4-ol (9.3%), and linalool (4.3%) as main components. This extract was also characterised by the highest antioxidant activity and the highest concentration of polyphenolic compounds. The reduced numbers of the main aphid predators, i.e., syrphid flies and ladybirds, in the treatments using extracts went hand in hand with lower prey availability; however, the predator–prey ratio in these treatments was more favourable than in the control. The effect of water extracts of tarragon in reducing BBA populations may, on the one hand, result from the presence of volatile compounds that act as repellents and aphicides; on the other hand, some of the components present in the extracts are known for their ability to attract predators, such as syrphids (linalool), ladybirds (terpinen-4-ol, linalool, eugenol) and lacewings (linalool, eugenol). The final result achieved by using D10-E may be the combined effect of the mixture of these components. It is worth noting that the maximum concentration used in this experiment for dried mass was 10%. At this concentration, no phytotoxic effect on the protected plant was observed. More concentrated extracts, which might be more effective in controlling BBA, were not tested, which opens up scope for further research.

This 3-year study allowed us to assess the consistency of treatment effects on both aphids and their predators across different years. The results confirmed the repeatability of the tested extracts’ effects. However, the economic effectiveness of the applied control scenarios was not evaluated. The need to ensure the availability of plant material for preparing the extracts, as well as the need to prepare them weekly, may increase labour costs. Furthermore, since the occurrence of aphids and predators is inextricably linked, it is not possible to definitively determine whether the final effect is the result of the extracts’ impact on aphids alone, their impact on the community of natural enemies, or both. Thus, future research on the use of tarragon extracts should focus on evaluating the cost–benefit ratio and investigating their direct impact on natural enemies. While this has been partially done in the case of ladybirds [20], there is still a lack of information regarding the impact on syrphid flies, other predators, and parasitoids. Plant-level measurements would also significantly enhance the practical value of future research and the potential application of tarragon extracts in agriculture.

## 4. Materials and Methods

### 4.1. Experimental

The field experiment was carried out between 2022 and 2024 at the Experimental Station of the University of Agriculture in Prusy, near Kraków (50.007′ N, 20.005′ E), Poland (warm temperate transitional climate). The average daily temperature and average precipitation during the study period were as follows: 2022—9.8 °C, 592.7 mm; 2023—10.4 °C, 751.7 mm; 2024—11.2 °C, 689.6 mm. The soil was a degraded chernozem, formed from loess, with a granulometric composition of silt loam. The soil pH in H_2_O was 6.56 and a humus content of 2.28%. The ‘Bizon’ variety of broad beans was used, sown at a standard row spacing of 50 cm. The individual plot area was 36 m^2^ (6 m × 6 m). The 2-metre gaps between the experimental plots were mechanically maintained as bare soil. Broad bean seeds were sown at the end of March (25 March 2022, 30 March 2023 and 31 March 2024). Within the rows, a spacing of 15 cm was kept between the broad bean seeds. The seeds were planted 6 cm deep in the soil. Weed control was carried out using mechanical methods.

The following experimental treatments were established:

- Broad beans protected with 2% concentration of dry mater extract (D2);
- Broad beans protected with 5% concentration of dry mater extract (D5);
- Broad beans protected with 10% concentration of dry mater extract (D10);
- Broad beans protected with 10% concentration of fresh mater extract (F10);
- Broad beans protected with 20% concentration of fresh mater extract (F20);
- Broad beans protected with 30% concentration of fresh mater extract (F30);
- Broad beans non—protected, distilled water used in the same amount as in the case of extract treatments (control);
- Broad beans subjected to standard chemical pest control practice (Ch).

The experiment was carried out in four repetitions in accordance with a randomised block design. In Ch treatment, the following chemical insecticides were used to protect crops, in accordance with conventional pest control practices: Deltam AL (SBM Développement SAS, Ecully, France, deltamethrin—0.0075 g, sprayed until the plants are completely covered with the liquid, as recommended on the label) against pea leaf weevils (when the first damage caused by those agrophages was noticed), Cyperkill Max 500 EC (Arysta LifeScience Benelux SPRL, Seraing, Belgium, cypermethrine, 500 g/L) to combat BBA (0.05 L/ha at the time of appearance of aphids on the broad bean plants, repeated after 10 days) and Aceptir 200 SE (Innvigo Sp. z o.o., Warsaw, Poland, acetamiprid—200 g/L) to combat the broad bean weevil (*Bruchus rufimanus* Boh.) (0.2l/ha, BBCH 50–89). For treatments involving the use of extracts, spraying was carried out once a week from the emergence stage (usually in late April and early May) until the end of the BBA occurrence period (usually around mid-June). A hand-held knapsack sprayer (Titan 12 knapsack sprayer, Marolex, Łomna, Poland) was used, fitted with an MR1.5 adjustable-angle nozzle (liquid flow rate 0.50 L/min), operating at a pressure of 0.4 MPa, with a total liquid volume of 300 L/ha.

### 4.2. Extract Preparation

*Artemisia dracunculus* plants grown at the University of Agriculture in Kraków, in south-eastern Poland, were used to prepare the extracts. The plants were grown at the same location and under the same soil and climatic conditions as the main field experiment. During the period when tarragon was being used to prepare extracts, no diseases or pests were observed on the plants; consequently, there was no need to apply chemical protection measures. Weeding was carried out by hand. The plants were not fertilised. For the dry-matter extracts, the harvested above-ground parts of the plants were first air-dried indoors, in a shaded, dry place, at room temperature for 7 days and then hand-chopped with secateurs into pieces approximately 1 cm long. In contrast, for the preparation of fresh-matter extracts, plants harvested on the same day were used; these were also fragmented, in the same way as the dried material, before being immersed in redistilled water.

The extracts were prepared at concentrations conventionally adopted as 2%, 5% and 10% for D-Es (dried plant material [g] + cold redistilled water [mL] in ratios of 2:100, 5:100 and 10:100) and 10%, 20% and 30% for F-Es (fresh plant material [g] + cold redistilled water [mL] in ratios of 10:100, 20:100 and 30:100). The doses were determined on the basis of previous experiments ([18] and preliminary studies), which demonstrated greater efficacy of extracts prepared from dry matter than from fresh plant material. The extracts were stored in the dark for 24 h, then filtered through filter paper and immediately used in the experiment.

### 4.3. BBA and Predator Abundance

During BBA monitoring (only the asexual population of *A. fabae* was studied [58]), the number of individual aphid morphological forms (wingless females, winged females, nymphs) was determined. Observations were conducted once every 3–4 days on 30 randomly selected and marked plants in each plot. They were initiated when the first migrating winged females were observed and lasted until the aphids had clearly ended their appearance (no live specimens were observed on the plants). Aphids were counted on the entire plant. Generally, when the number of aphids was less than 100, all individuals were counted precisely; when this number increased, the number of aphids was estimated using the method described previously [23]. Briefly, in the case of aphids feeding on the apex, all individuals were carefully counted. In the case of aphids feeding on the shoot, all individuals were counted within a 1 cm section, and this value was then multiplied by the length of the colony on the shoot. The same procedure applied to aphids feeding on pods. In the case of aphids feeding on leaves or flowers, all individuals on a single leaf (or flower) were counted and multiplied by the number of leaves (or flowers) infested by aphids. During counting, the leaves were gently turned over. Care was taken during counting to ensure that no aphids were shaken off the plant.

The occurrence of aphid predators was assessed simultaneously with the presence of aphids, on the same plants selected for the assessment of BBA occurrence, and on the same dates. The occurrence and abundance of predators were analysed at individual developmental stages (eggs, larvae and pupae in the case of syrphids; eggs, larvae, pupae and adults in the case of ladybirds; eggs and larvae in the case of lacewings; and larvae and adults in the case of anthocorids and spiders). Lacewings, anthocorids and spiders were presented in Section 2 as a combined group labelled ‘other predators’. All Syrphidae pupae found were transferred to the laboratory and kept in Petri dishes until the adults emerged (22–24 °C, relative humidity 70%). The hatched adults were identified to species level [59,60].

### 4.4. Yield Assessments of Broad Beans

After completing the observations on the occurrence of BBA and predators, 20 randomly selected whole plants were collected from each plot (excluding plants from the outer rows), and the seed weight of each plant was measured in the laboratory. Seed yield was calculated by multiplying the average seed weight per plant in a given plot by the number of plants in that plot, and the seed yield was then converted to a yield per 1 m^2^.

### 4.5. HS-SPME-GC-FID-MS Analysis of Volatile Compounds

For analysis of volatile compounds from *A. dracunculus* FH and DH as well as from water extracts, the HS-SPME-GC-FID-MS technique was used. Sampling was carried out using SPME grey fibre 50/30 μm DVB/CAR/PDMS, stableflex 2 cm (Supelco, Bellefonte, PA, USA). Then, 0.5 g of dried herb or 1.0 g fresh herb or 1 mL of each water extract was introduced to 15 mL Amber vial with hole cap PTFE/Silicone septa (Supelco, Bellefonte, PA, USA). Incubation and extraction lasted 30 min at 60 °C each. Every analysis was performed in two repetitions using Trace GC Ultra gas chromatograph coupled with DSQ II mass spectrometer (Thermo Electron Corporation, Waltham, MA, USA). The operating conditions were as follows: non-polar capillary column Rtx-1ms (60 m × 0.25 mm, 0.25 m film thickness); programmed temperature: 50 (3 min)–300 °C, 4 °C/min, injector (SSL) temperature 280 °C, detector (FID) temperature 300 °C, transfer line temperature 250 °C, carrier gas: helium, flow with constant pressure: 200 kPa, split ratio 1:20. The mass spectrometer parameters: ion source temperature: 200 °C, ionization energy 70 eV (EI), scan mode: full scan, mass range 33–420. The percentages of constituents were computed from the GC peak area without using a correction factor. The identification of the constituents was based on a comparison of their mass spectra and linear retention indices (RI, non-polar column), determined with reference to a series of n-alkanes C8-C24 with literature data [61] and computer libraries NIST 2011, and MassFinder 4.1.

### 4.6. Antioxidant Activity of A. dracunculus Extracts

Antioxidant activity was determined using modified method of Brand-Williams et. al. [62] with the 2,2-diphenyl-1-picrylhydrazyl (DPPH). A DPPH solution was prepared at a concentration of 0.11 mM in ethanol (96%, pure p. a.). The control sample (A_0_) was prepared by mixing 3 mL of DPPH solution and 20 µL of water. The test samples were prepared by mixing 3 mL of DPPH solution with 20 µL of the tested extract of *A. dracunculus*. Next, samples were incubated at room temperature for 30 min in the dark. Afterwards, absorbance of prepared samples was measured at a wavelength of 517 nm against ethanol (blank) using Nicolet Evolution 300 dual-beam UV-VIS spectrophotometer (Thermo Electron Corporation). Each measurement was repeated three times, and the average value was calculated. To prepare standard curve, 2 mM concentration of Trolox was prepared in ethanol (96%, pure p. a.). The following dilutions of this solution (2.0; 1.25; 1.0; 0.5; 0.25; 0.1 mM) were prepared. The preparation of the solutions for the calibration curve and the performance of the spectroscopic analysis were analogous to those for the tested extracts. The antioxidant activity of the *A. dracunculus* extracts was calculated based on the calibration curve equation (y = −0.503x + 1.2951; R^2^ = 0.996) and expressed as the Trolox equivalent antioxidant capacity (TEAC, mM). The results were also presented as the DPPH radical scavenging activity (RSC, %), according to the following equation:

RSC, % = 100 (A_0_ − A)/A_0_

where: A_0_—average absorbance of the control sample

A—average absorbance of the test sample

### 4.7. The Total Content of Polyphenols (TCP) in A. dracunculus Extracts

Here, 0.5 mL of aqueous extract from *A. dracunculus* plant material (dried or fresh) was added to a 25 mL volumetric flask; then, 0.25 mL of Folin-Ciocalteu solution was added and mixed gently. Next, 2.5 mL of 20% water solution of sodium carbonate was added and diluted with distilled water. Mixture was stirred vigorously and incubated in a dark place at room temperature for 30 min. Absorbance was measured at a wavelength of 750 nm using a VIS-7220G spectrophotometer (Beijing Beifen-Ruili Analytical Instrument Co., Ltd., Beijing, China). As a blank test, pure distilled water was tested, and 25–1000 mg/L aqueous solution of gallic acid was used as a standard. The results were expressed in mg of gallic acid equivalents per g of extract (mg GAE/g). For the prepared standard curves, R^2^ ≥ 0.9975, measurement was performed three times, and the results were averaged.

### 4.8. Statistical Analysis

The obtained data (number of aphids and individual stages of predators, yield) were checked for normality (Shapiro–Wilk test with Lilliefors correction) and, when necessary, log-transformed. Then, they were analysed using STATISTICA 13.0 software. The significance of differences between the treatments was tested first by two-factor ANOVA (treatment and year of study as factors). Because each of the parameters analysed was significantly affected by the year of study (Tables S1, S4 and S5), the data from each year were then analysed separately. One-factor ANOVA (treatment as factor) in each year was performed. This approach was adopted to ensure greater clarity of the results. The data showing the main effects—the effects of treatment, year and the interaction between these factors—are presented in Tables S2 and S6. In order to examine the effect of the observation date, a two-factor ANOVA was carried out for each of the study seasons (treatment and date as factors). As, in most cases, a significant effect of the observation date on the parameters under investigation was observed (Table S3) and there was no significant interaction between the date of the observation and the treatment, it was subsequently decided to carry out a one-factor ANOVA (treatment as factor) for each observation date separately. The means were differentiated by Fisher’s LSD test at a level of *p* < 0.05. The analysis of aphid and predator densities was based on the average values from individual plots, i.e., from 30 plants in each plot. Each treatment comprised 4 plots (replicates). In order to determine the quantitative relationships between the components of the BBA predator community, numerical predator–prey ratios were also calculated (average data from individual plots were used; the total number of aphids was divided into three equal parts, and each of these parts (the number of aphids) was then divided by the number of a specific predator—larvae of the family Syrphidae, larvae of the family Coccinellidae or an adult of the family Coccinellidae). The significance of differences between the treatments in this case was tested first by two-factor ANOVA (treatments and year of study as factors). Because most parameters analysed were significantly affected by the year of study (Table S3), the data from each year were then analysed separately, and one-factor ANOVA (treatment as factor) was performed. The Pearson correlation coefficient was also calculated for aphids and their main predators, i.e., Syrphidae and Coccinellidae. Principal Component Analysis assessed the relationships between aphid density, predator density and extracts used.

The results of antioxidant activity and total content of phenolic compounds were presented as means ± SD with n = 3 replicates. Assumptions regarding normal distribution in individual groups and homogeneity of variance were fulfilled. The analysis was performed using an ANOVA test followed by Tukey’s post hoc test at significance level 0.05. Statistical analyses were performed using Freeware Jamovi Software version 2.6.44.

## 5. Conclusions

The aqueous extract prepared from dried *A. dranunculus* at a concentration conventionally set at 10% proved to be effective in controlling BBA, whilst no adverse effects were observed on their predators. The ease of preparation and availability of the material make it ideal for use on organic farms or as part of integrated pest management. Further research could focus on the possibilities of commercially manufacturing the extract as a ready-to-use product. It would also be advisable to determine the direct impact of the extracts studied on non-target organisms.

## Figures and Tables

**Figure 1:**
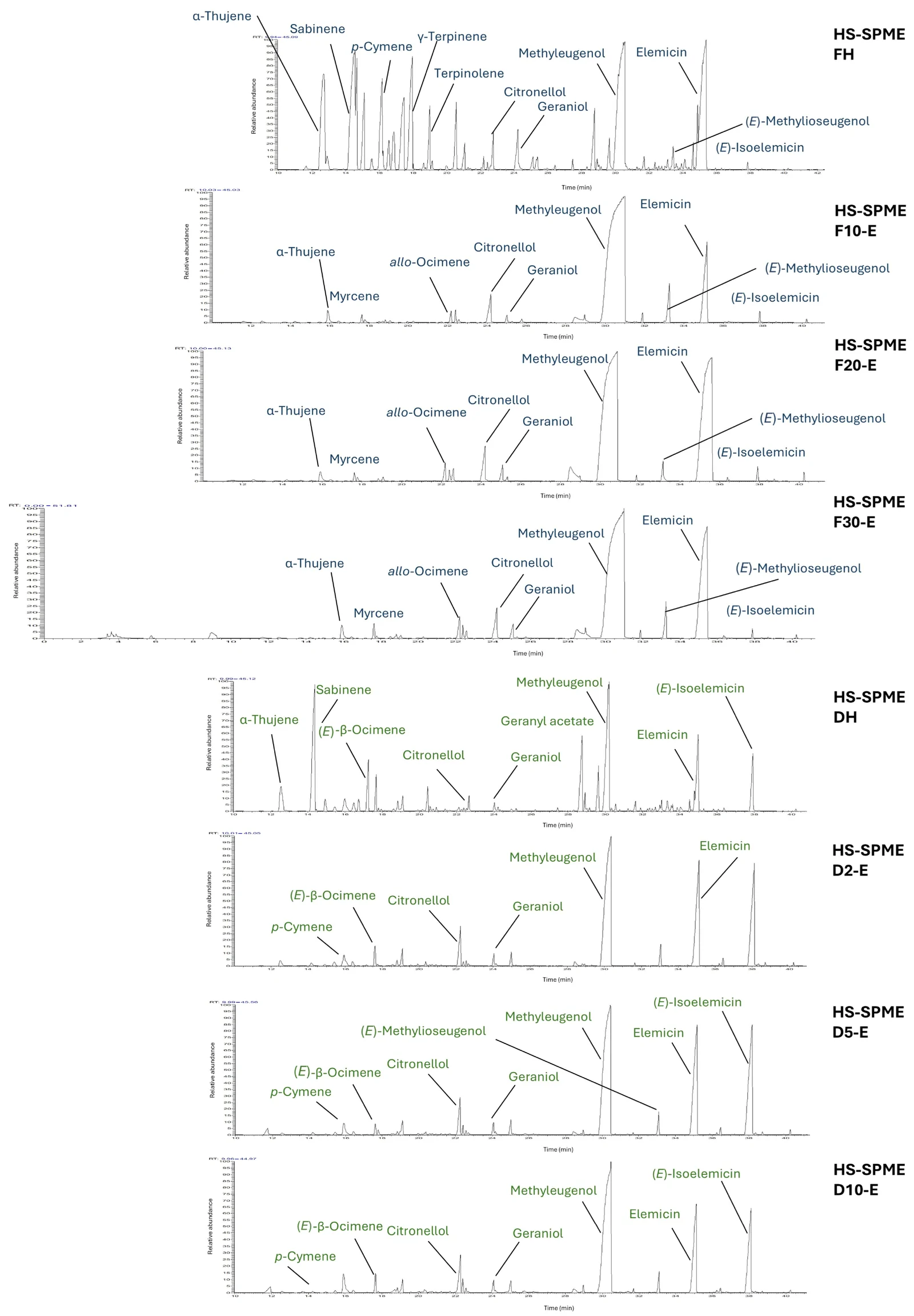
Chromatographic profile of volatile compounds isolated from *Artemisia dracunculus* head space from fresh (FH) and dried herb (DH) and water extracts from fresh and dried plant material. The y-axis represents the peak intensity. The x-axis represents the retention time. The area under the peak is considered the concentration of the component. For explanation, see Table 1.

**Figure 2:**
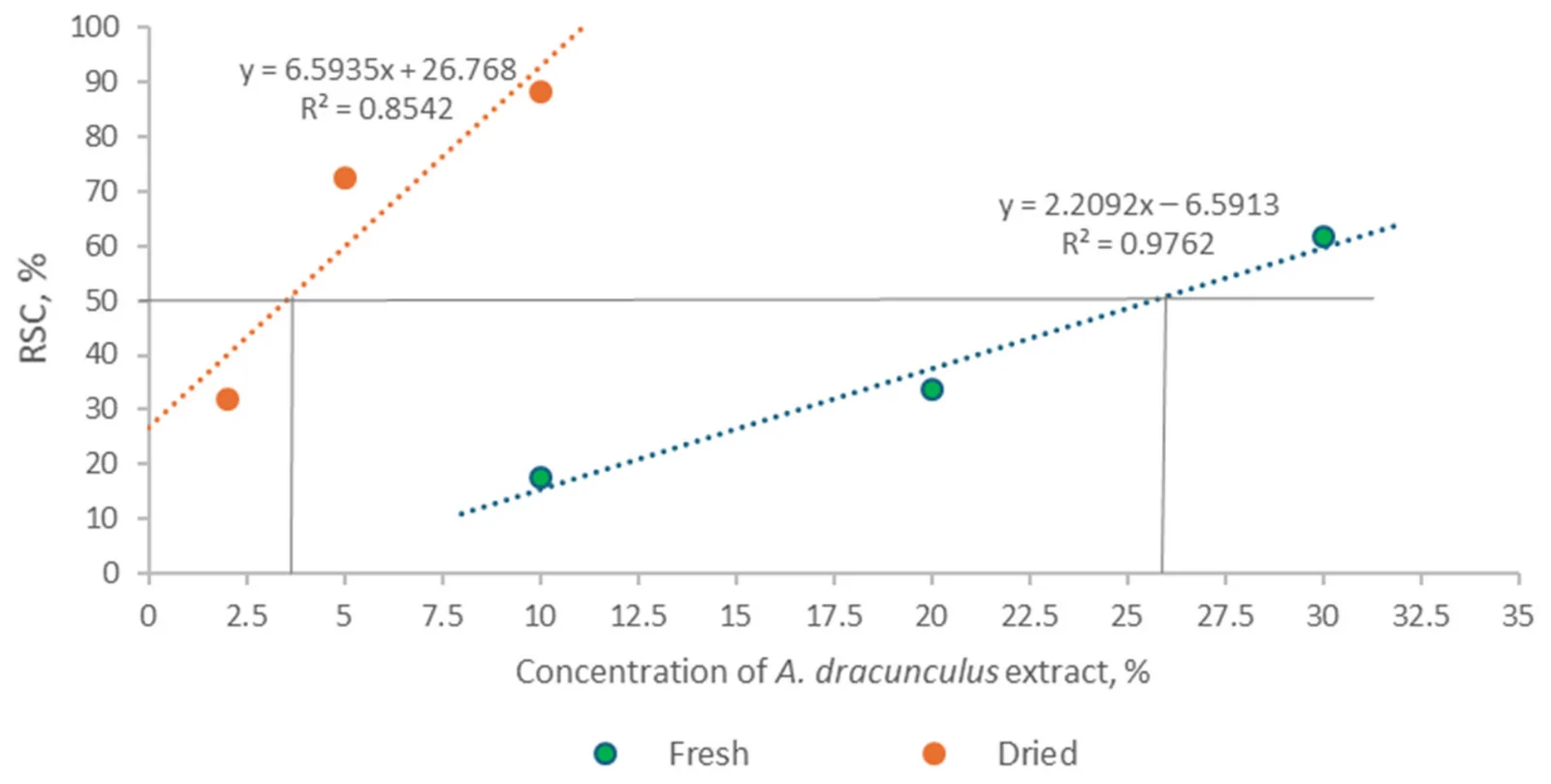
Radical scavenging activity (RSC) dependence on *Artemisia dracunculus* extract concentration—determination of EC_50_ values. The dots represent the percentage concentrations of tarragon extract (dried and fresh) used to determine antioxidant activity.

**Figure 3:**
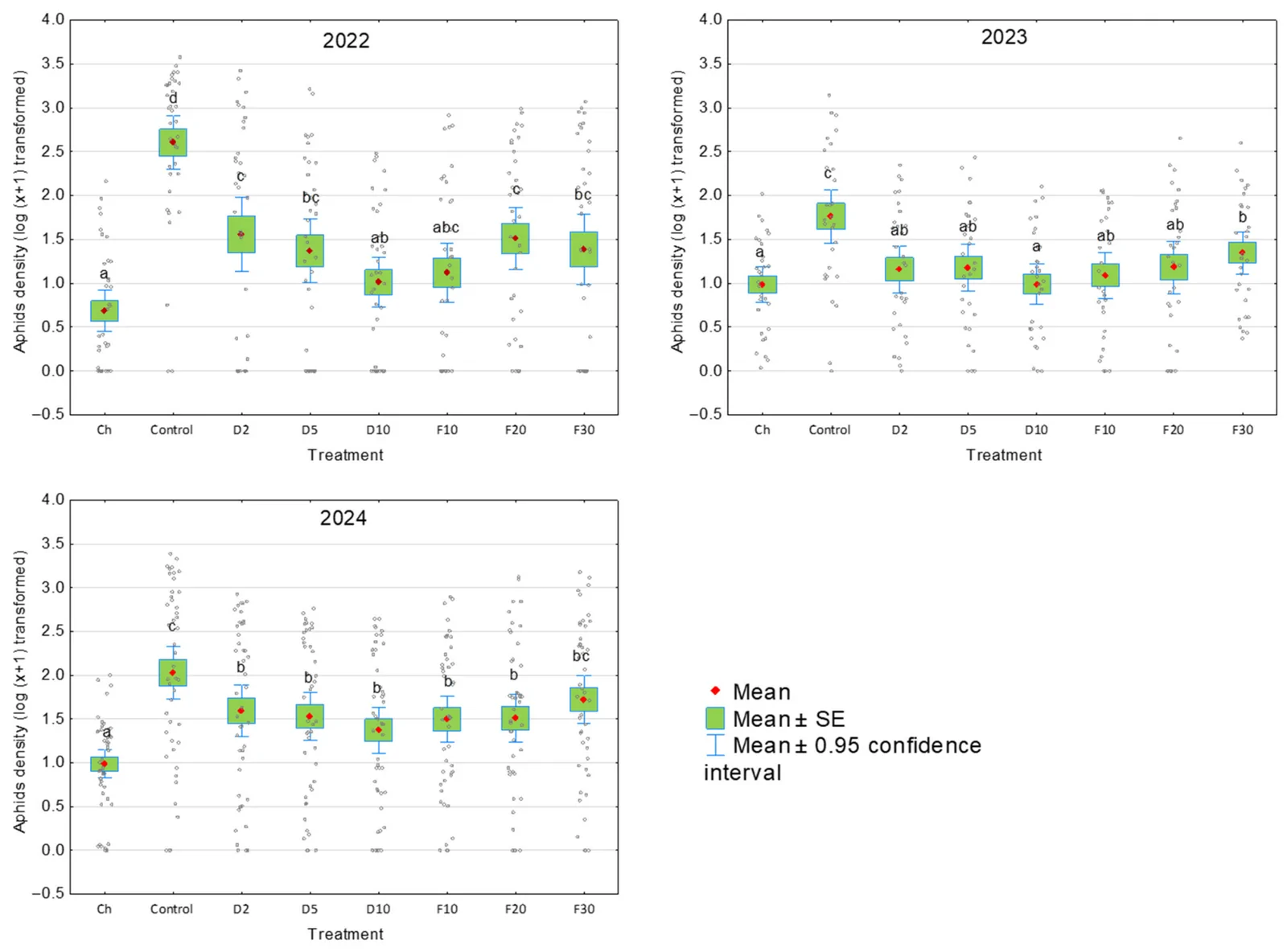
*Aphis fabae* density on broad beans in the 2022–2024 seasons. Means marked with the same letters do not differ significantly according to LSD test at *p* < 0.05. ‘x’ on the y-axis represents the average number of aphids per plant in a given plot, grey dots represent the average values from individual plots over the entire observation period. Treatments: Ch—broad beans subjected to standard chemical pest control practice; control—broad beans non-protected, distilled water used in the same amount as in the case of extract treatments; D2—broad beans protected with 2% concentration of dry mater extract; D5—broad beans protected with 5% concentration of dry mater extract; D10—broad beans protected with 10% concentration of dry mater extract; F10—broad beans protected with 10% concentration of fresh mater extract; F20—broad beans protected with 20% concentration of fresh mater extract; F30—broad beans protected with 30% concentration of fresh mater extract.

**Figure 4:**
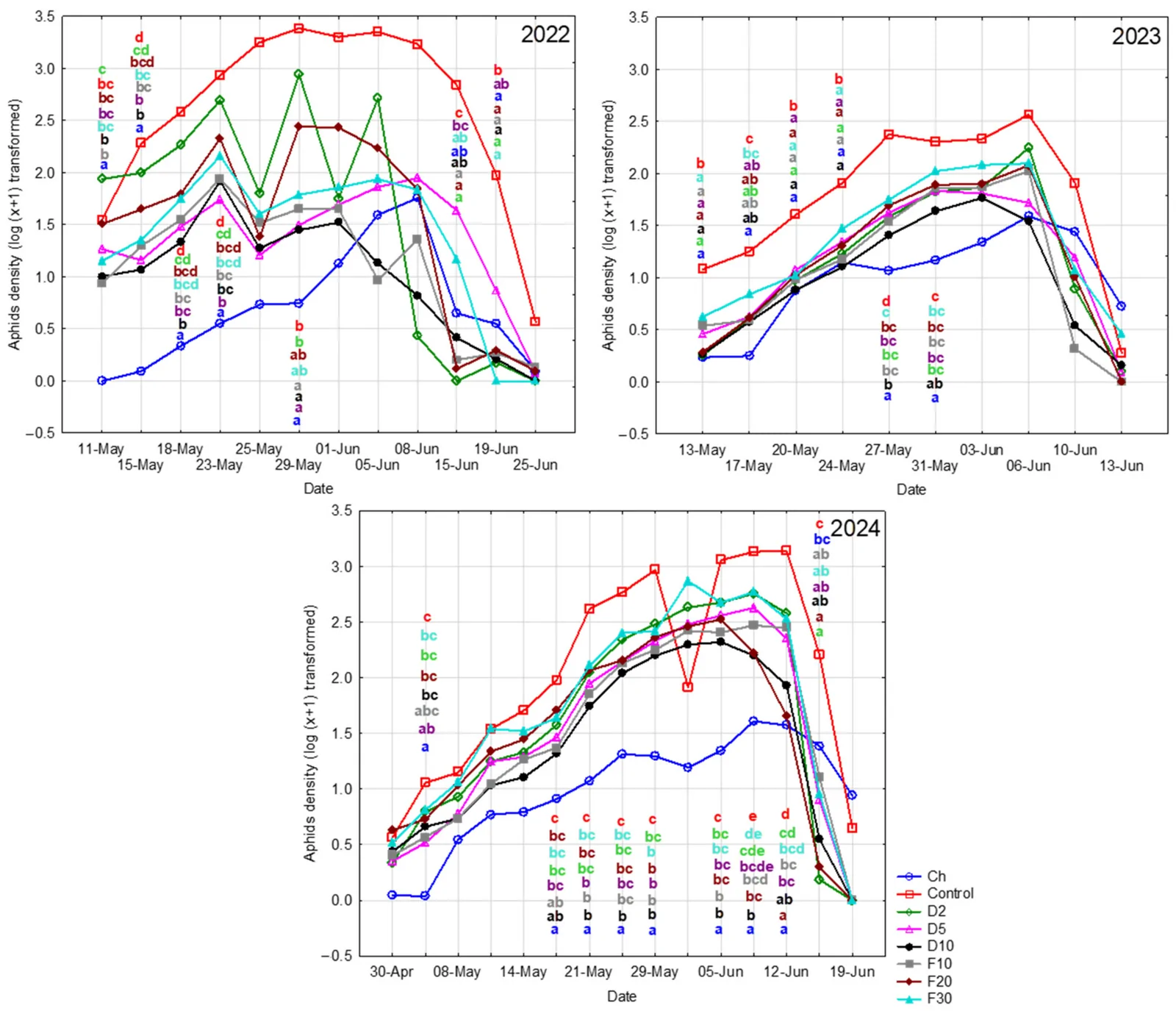
*Aphis fabae* density on broad beans at individual days of observations in the 2022–2024 seasons. Means marked with the same letters for each day separately do not differ significantly according to LSD test at *p* < 0.05. Letters were used only when there were significant differences between treatments. ‘x’ on the y-axis represents the average number of aphids per plant in a given plot. For treatments, see Figure 3.

**Figure 5:**
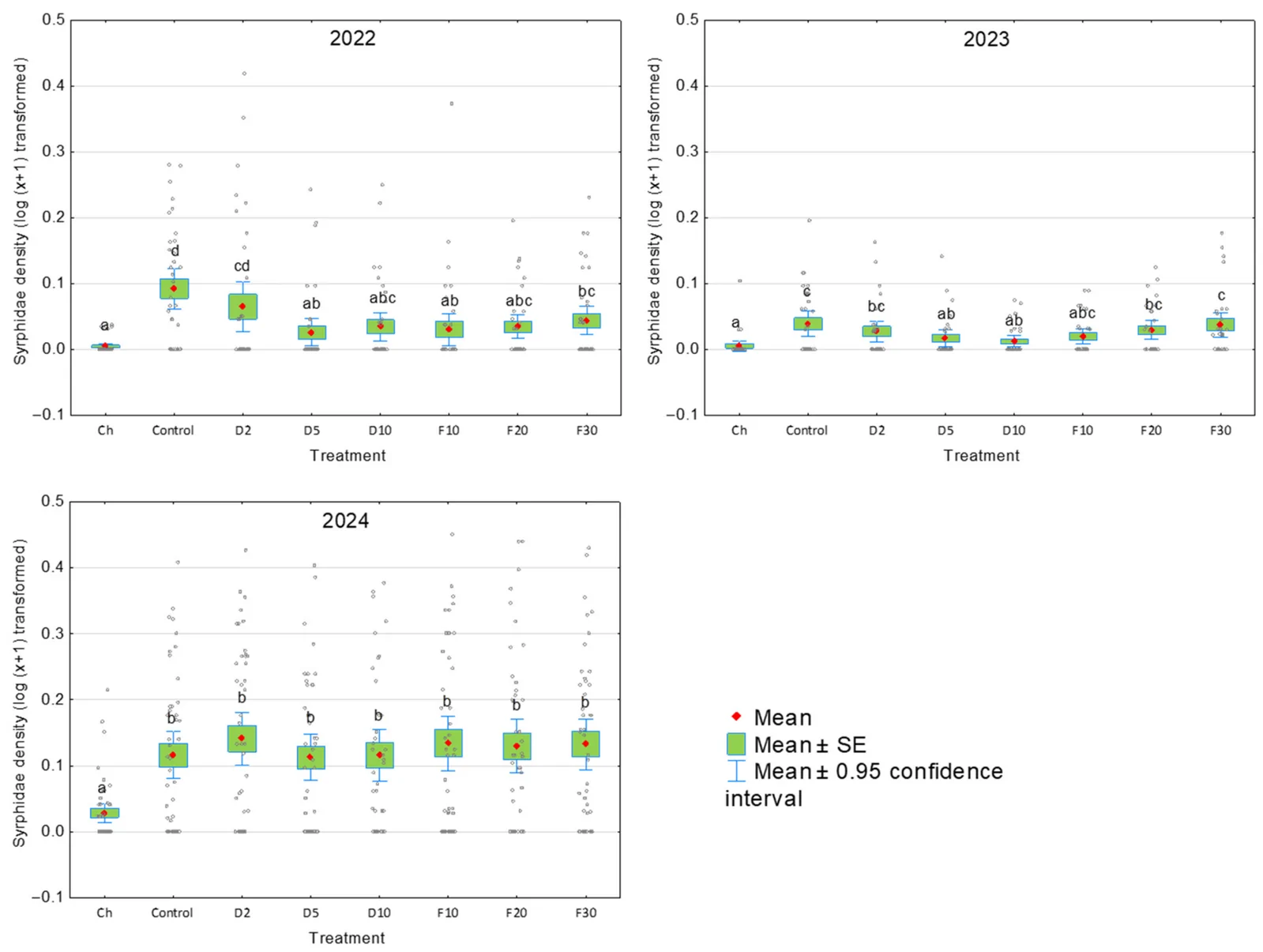
Syrphidae density on broad beans in the 2022–2024 seasons. Means marked with the same letters do not differ significantly according to LSD test at *p* < 0.05. ‘x’ on the y-axis represents the average number of Syrphidae per plant in a given plot, grey dots represent the average values from individual plots over the entire observation period. For treatments, see Figure 3.

**Figure 6:**
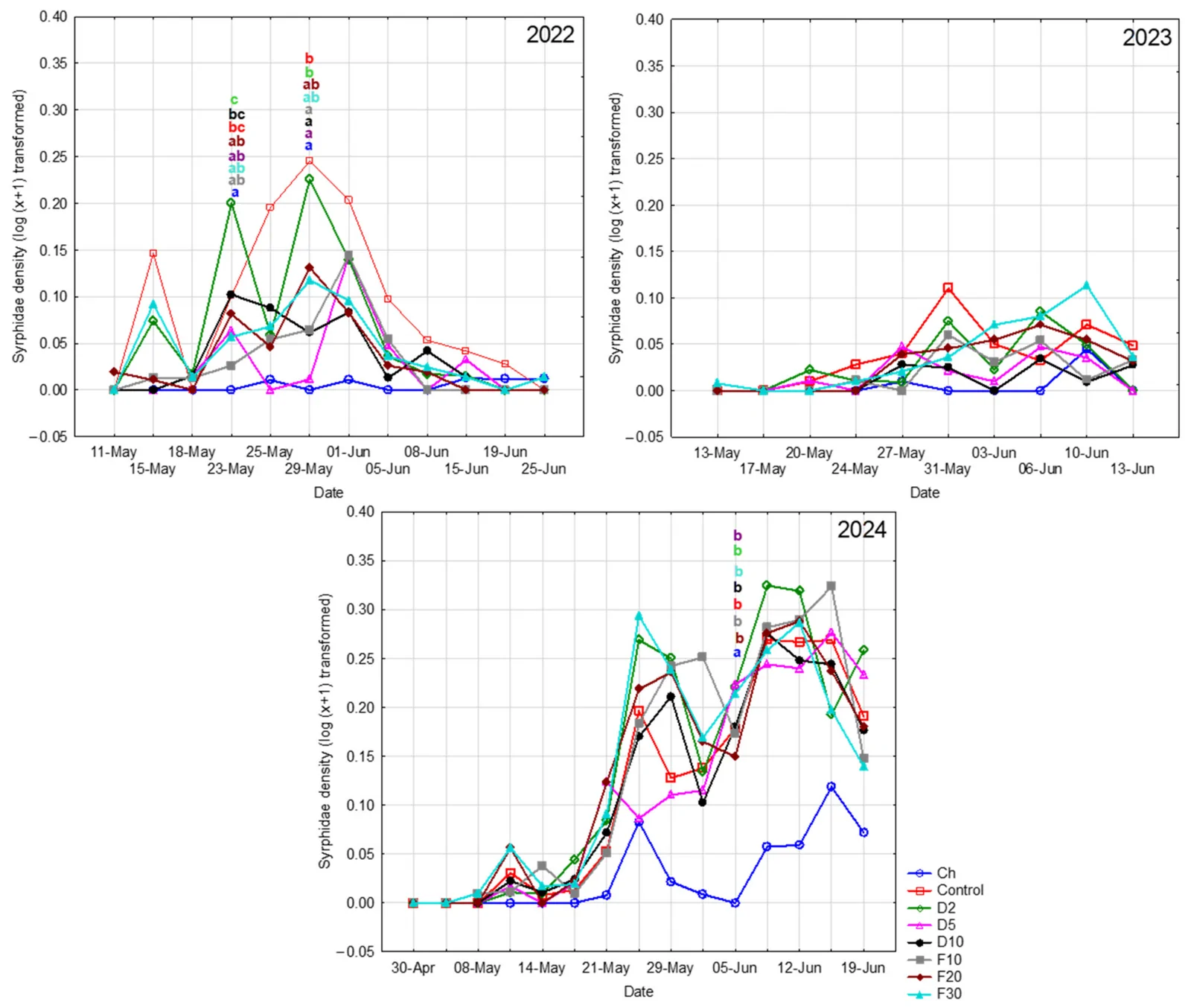
Syrphidae density on broad beans at individual days of observations in the 2022–2024 seasons. Means marked with the same letters for each day separately do not differ significantly according to LSD test at *p* < 0.05. Letters were used only when there were significant differences between treatments. ‘x’ on the y-axis represents the average number of Syrphidae per plant in a given plot. For treatments, see Figure 3.

**Figure 7:**
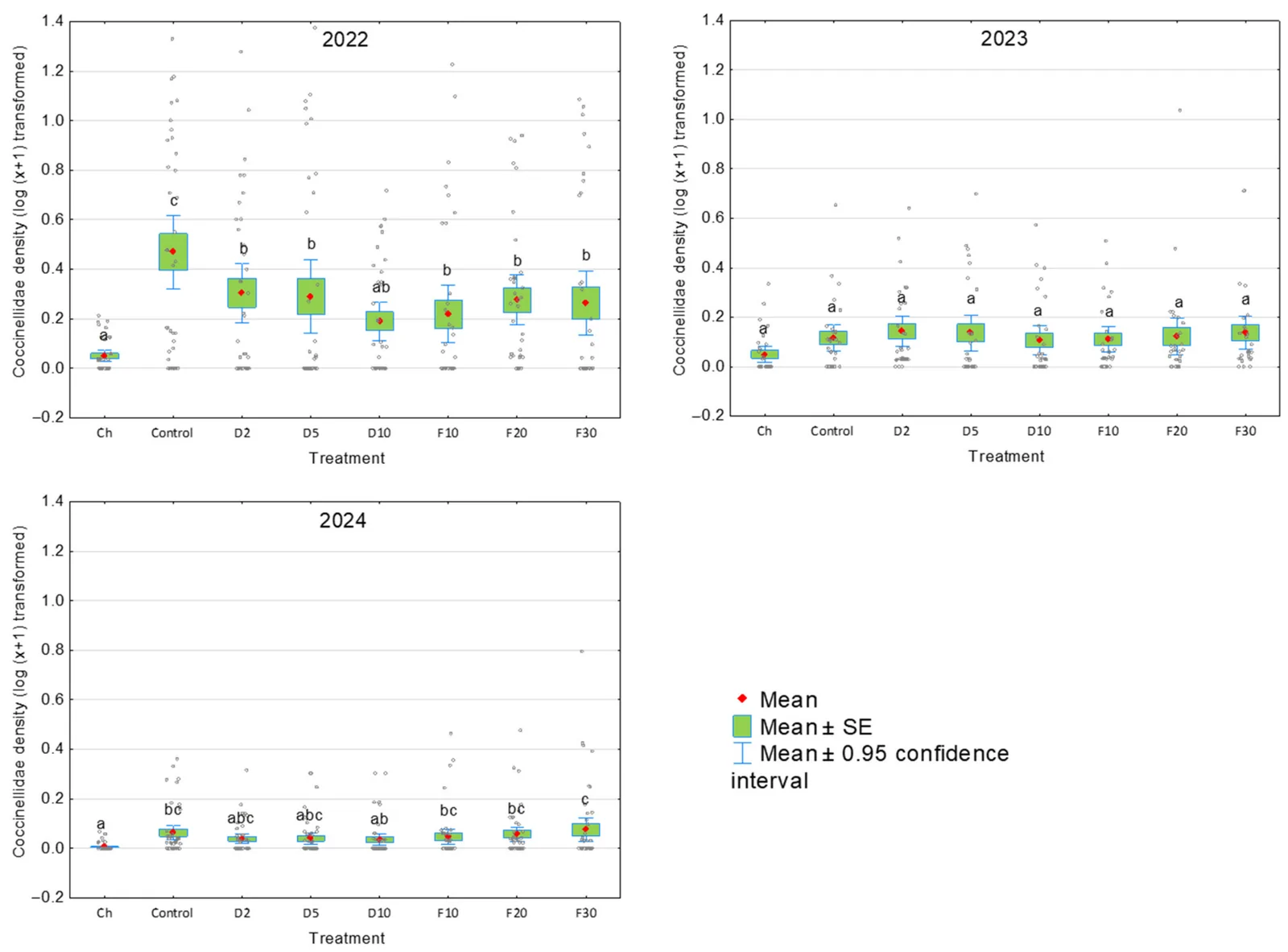
Coccinellidae density on broad beans in the 2022–2024 seasons. Means marked with the same letters do not differ significantly according to LSD test at *p* < 0.05. ‘x’ on the y-axis represents the average number of Coccinelidae per plant in a given plot, grey dots represent the average values from individual plots over the entire observation period. For treatments, see Figure 3.

**Figure 8:**
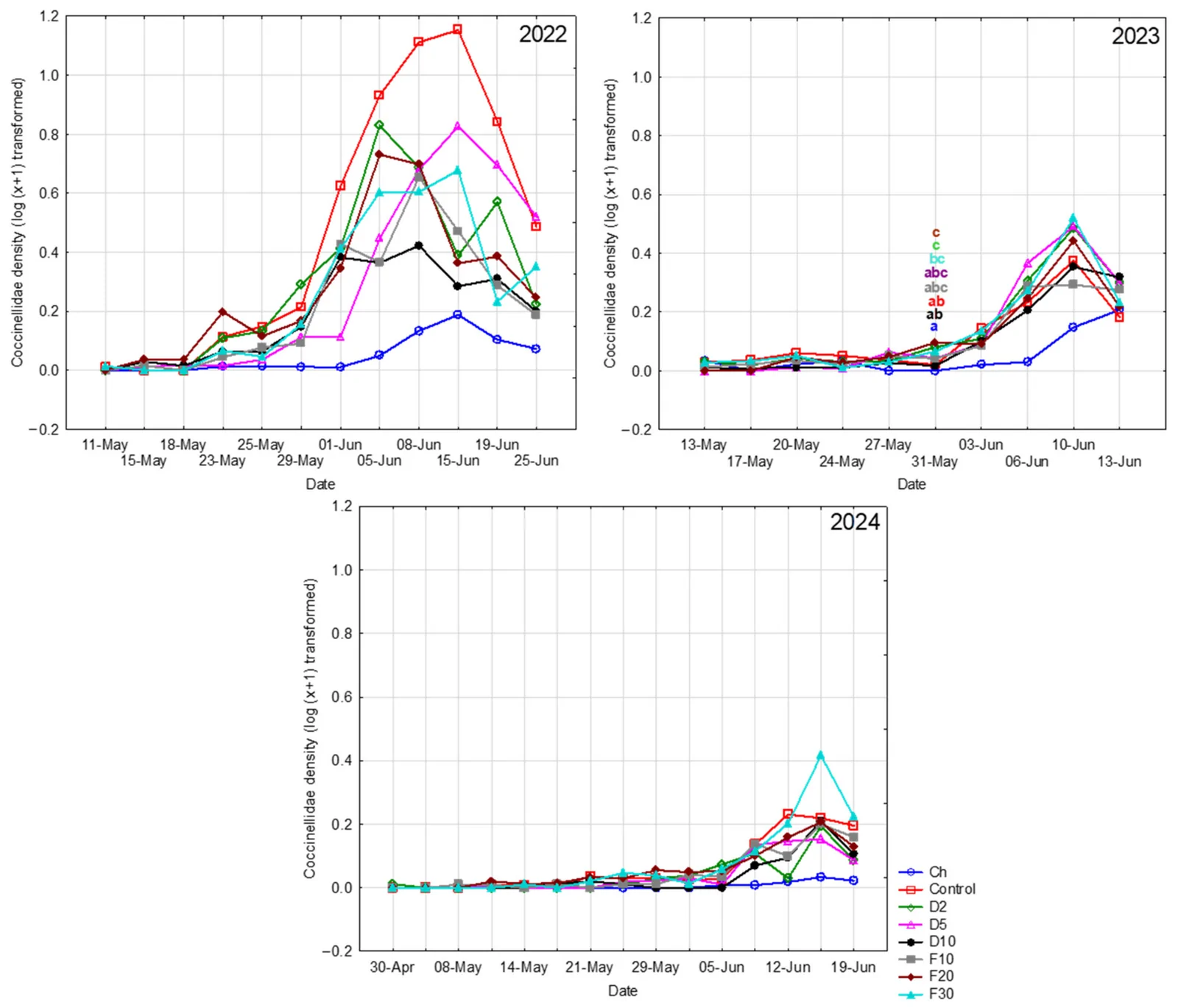
Coccinellidae density on broad beans at individual days of observations in the 2022–2024 seasons. Means marked with the same letters for each day separately do not differ significantly according to LSD test at *p* < 0.05. Letters were used only when there were significant differences between treatments. ‘x’ on the y-axis represents the average number of Coccinelidae per plant in a given plot. For treatments, see Figure 3.

**Figure 9:**
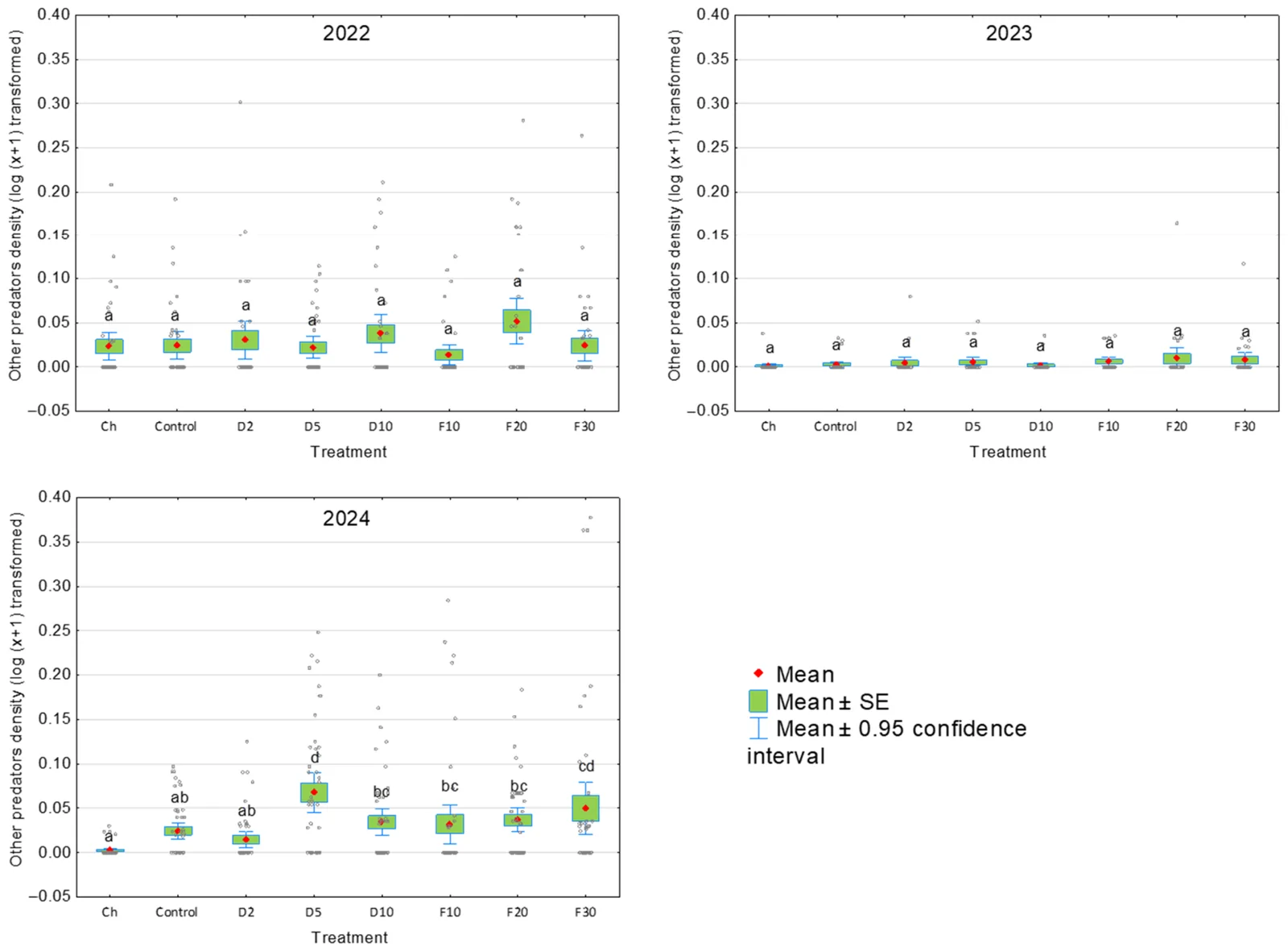
Other predators’ density on broad beans in the 2022–2024 seasons. Means marked with the same letters do not differ significantly according to LSD test at *p* < 0.05. ‘x’ on the y-axis represents the average number of other predators per plant in a given plot, grey dots represent the average values from individual plots over the entire observation period. For treatments, see Figure 3.

**Figure 10:**
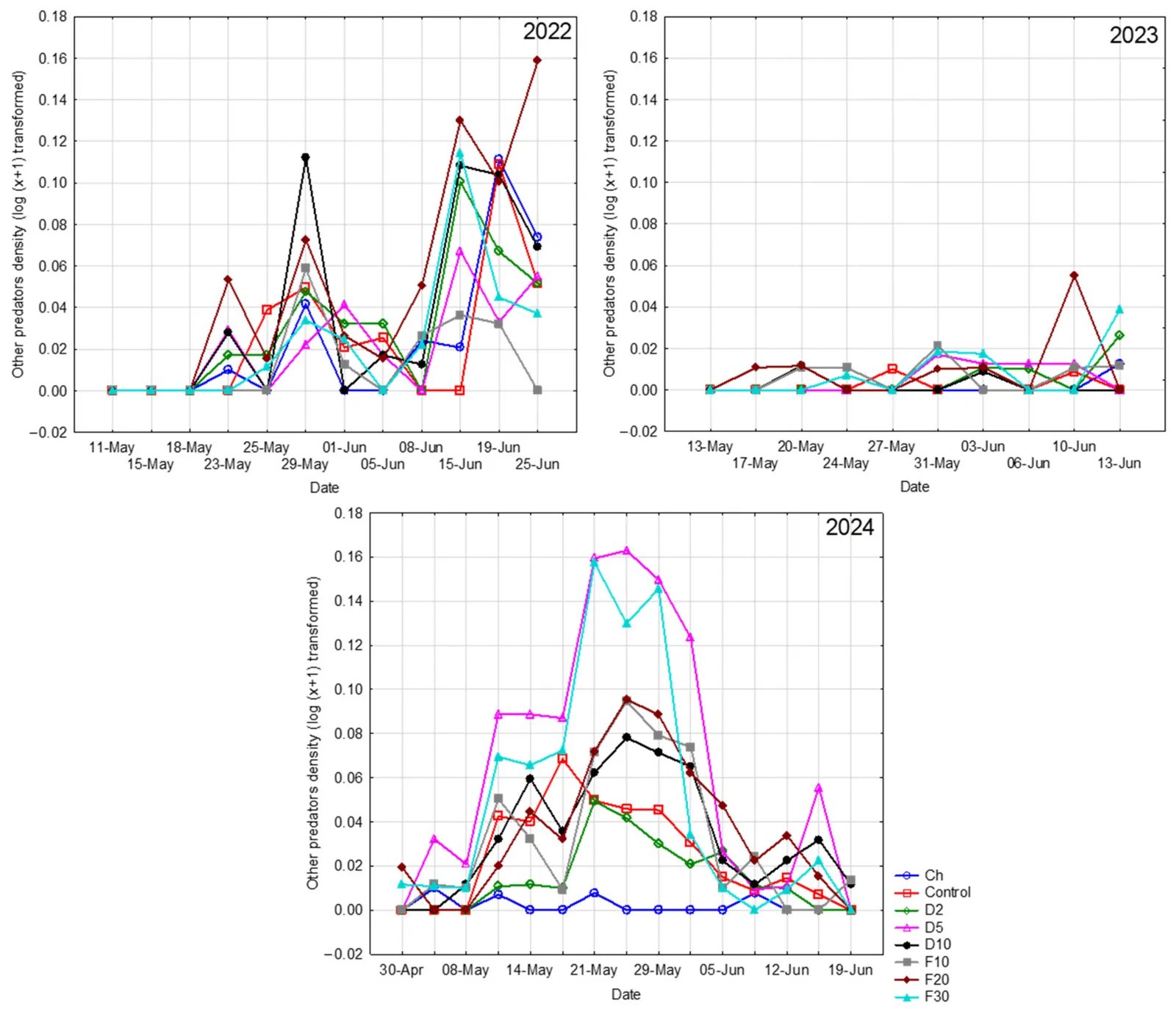
Other predators’ density on broad beans at individual days of observations in the 2022–2024 seasons. Means do not differ significantly according to LSD test at *p* < 0.05. ‘x’ on the y-axis represents the average number of other predators per plant in a given plot. For treatments, see Figure 3.

**Figure 11:**
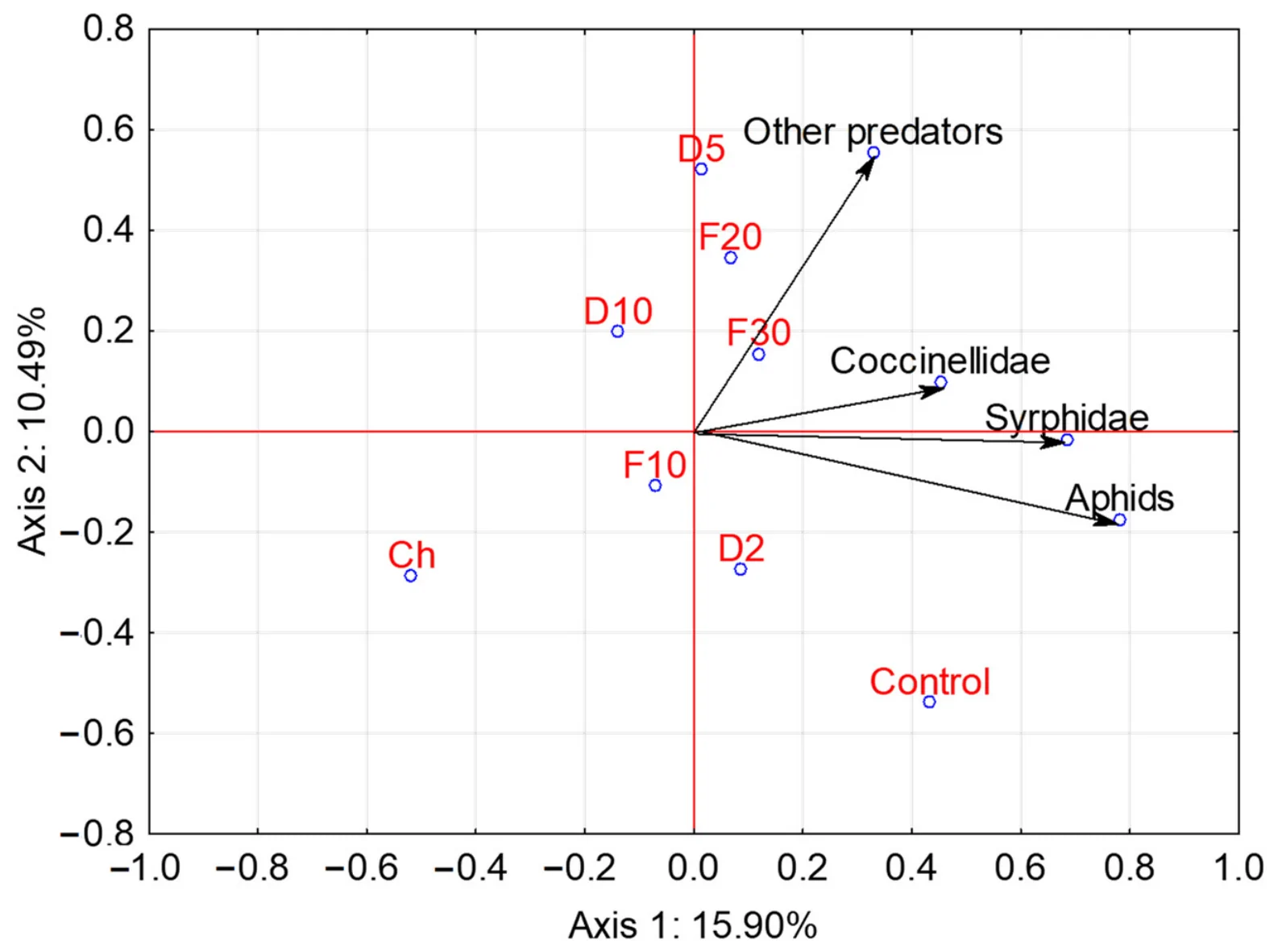
PCA for aphids, predator abundance and treatments. For treatments, see Table 3.

**Figure 12:**
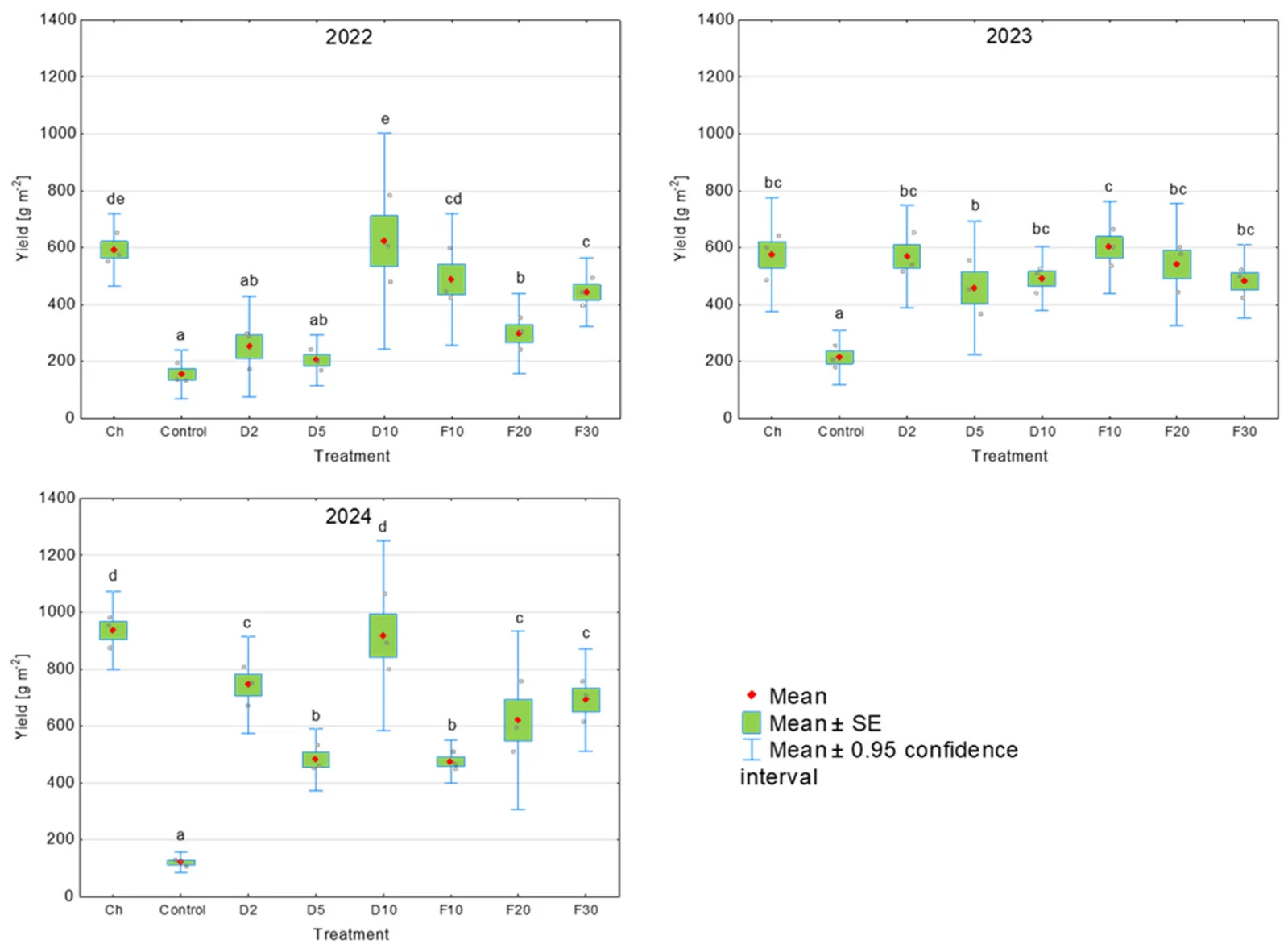
Broad bean seed yield [g m^−2^] in the 2022–2024 seasons. Means marked with the same letters do not differ significantly according to LSD test at *p* < 0.05. For treatments, see Figure 3.

**Table 1:** Chemical composition of volatile compounds from head space of fresh and dried herb and water extracts received from *Artemisia dracunculus.*

Compound	RI lit	RI exp	FH	DH	F10-E	F20-E	F30-E	D2-E	D5-E	D10-E
			[%]							
α-Thujene	932	920	11.1	5.0	0.6	0.7	0.4	-	-	-
Benzyl aldehyde	941	937	-	-	t	0.1	0.1	0.1	0.1	0.2
α-Pinene	934	927	0.7	0.1	0.3	t	0.2	1.2	0.8	0.8
Sabinene	970	966	9.4	18.8	0.5	0.5	0.5	0.6	0.7	0.7
β-Pinene	974	968	5.3	0.1	-	-	-	-	-	-
Myrcene	983	980	4.8	2.3	0.2	0.3	0.2	0.4	0.3	0.4
α-Phellandrene	998	998	0.6	0.7	0.3	0.3	0.3	1.0	0.7	0.9
α-Terpinene	1013	1013	0.3	t	0.5	0.5	0.4	1.7	1.2	1.9
*p*-Cymene	1116	1009	4.6	t	0.5	t	0.4	0.7	0.6	1.3
β-Phelleandrene	1025	1017	0.9	t	0.7	0.6	0.6	0.9	0.9	1.0
Limonene	1025	1018	0.9	1.5	t	0.1	-	-	-	-
(*Z*)-β-Ocimene	1025	1025	3.2	1.9	t	t	t	0.1	t	0.1
(*E*)-β-Ocimene	1041	1036	7.6	6.7	0.1	0.2	0.1	0.2	0.1	0.2
γ-Terpinene	1051	1047	5.9	3.1	2.0	2.1	2.9	3.0	2.4	3.6
*trans*-Sabiene hydrate	1053	1051	0.1	0.3	0.7	0.9	0.8	0.3	0.7	0.4
*o*-Cymenene	1072	1070	t	0.2	-	-	-	0.1	t	t
Nonan-2-one	1074	1075	0.3	t	0.3	0.1	0.5	0.1	0.1	0.2
*p*-Cymenene	1074	1075	t	t	t	t	t	0.2	0.2	0.3
Terpinolene	1082	1078	2.6	1.1	0.5	0.6	0.7	0.8	0.6	0.9
*cis*-Sabinene hydrate	1084	1081	0.1	0.3	0.1	0.1	0.1	t	t	t
Linalool	1085	1084	0.3	1.9	1.0	1.1	1.0	2.9	3.2	4.3
*cis*-Rose oxide	1100	1099	t	t	0.1	t	t	0.2	0.1	0.2
Methyl octanoate	1100	1108	0.2	0.1	0.1	t	0.1	0.3	0.3	0.2
*allo*-Ocimene	1113	1123	2.5	2.1	-	-	-	0.5	0.4	0.4
Camphor	1123	1125	t	t	t	t	t	0.3	0.2	0.4
Benzyl acetate	1134	1135	t	t	t	t	t	0.1	0.1	0.2
Citronellal	1135	1136	1.1	-	0.1	0.1	0.1	-	-	-
Borneol	1150	1153	t	t	0.1	0.1	0.1	0.1	0.1	0.1
Terpinen-4-ol	1164	1163	0.5	0.5	3.6	4.0	5.0	7.3	6.8	9.3
Methyl salicylate	1170	1170	t	t	0.3	0.8	0.3	0.2	0.2	0.2
α-Terpineol	1176	1172	0.1	0.2	0.5	2.5	1.7	0.6	0.5	0.7
Estragol	1175	1176	1.2	1.0	0.2	t	t	0.3	0.3	0.3
Myrtenol	1178	1777	-	-	t	t	t	0.1	0.1	0.1
γ-Terpineol	1188	1191	-	-	-	0.1	0.1	-	-	-
Citronellol	1210	1210	1.1	1.0	6.3	8.0	8.0	1.8	2.4	3.6
Nerol	1214	1216	1.1	t	0.7	0.9	t	t	t	t
Geraniol	1235	1232	0.7	0.3	2.1	4.5	4.3	2.5	2.7	3.8
Linalyl acetate	1239	1235	t	t	t	t	t	t	0.1	0.1
Geranial	1243	1236	0.3	t	0.2	0.9	0.3	0.1	0.1	0.1
*trans*-Anethol	1262	1265	t	t	-	-	-	t	0.1	t
Thymol	1267	1265	t	t	t	t	t	t	t	t
Bornyl acetate	1270	1272	t	0.1	t	t	t	0.1	0.1	0.1
Carvacrol	1278	1272	-	-	t	t	t	t	t	t
Citronellic acid	1300	1302	0.3	0.2	-	-	-	-	-	-
Citronellyl acetate	1337	1335	2.6	8.0	-	-	-	-	-	-
Eugenol	1331	1332	t	t	1.2	3.0	2.4	0.7	0.5	1.3
α-Terpinyl acetate	1335	1341	-	0.1	-	-	-	0.2	0.2	0.1
Bicycloelemene	1338	1345	0.4	1.1	-	-	-	-	-	-
α-Cubebene	1355	1352	-	0.2	-	-	-	t	t	t
Geranyl acetate	1361	1361	1.3	4.6	-	-	-	0.1	t	t
Methyleugenol	1369	1378	17.6	16.6	55.7	39.2	38.2	39.1	39.4	27.7
β-Bourbonene	1386	1384	t	t	-	-	-	-	-	-
α-Isocomene	1389	1387	t	0.5	-	-	-	-	-	-
β-Cubebene	1389	1391	t	0.1	-	-	-	-	-	-
β-Isocomene	1411	1412	0.1	t	-	-	-	-	-	-
α-Gurjunene	1413	1416	t	t	-	-	-	-	-	-
(*E*)-β-Caryophyllene	1421	1424	0.5	0.1	-	-	-	-	-	-
(*Z*)-Methylisoeugenol	1422	1430	t	0.2	0.8	0.6	0.6	0.2	0.2	0.4
β-Copaene	1430	1433	0.1	0.2	-	-	-	-	-	-
Aromadendrene	1443	1444	0.2	0.1	-	-	-	-	-	-
Cadina-3,5-diene	1448	1446	0.1	0.2	-	-	-	-	-	-
(*E*)-β-Farnesene	1446	1450	0.1	t	-	-	-	-	-	-
α-Humulene	1455	1456	0.1	0.3	-	-	-	-	-	-
ε-Muurolene	1455	1461	0.1	0.4	-	-	-	-	-	-
(*E*)-Methylisoeugenol	1466	1463	0.2	0.5	4.3	2.6	3.9	2.0	2.0	2.7
γ-Curcumene	1473	1471	t	t	-	-	-	-	-	-
γ-Muurolene	1474	1476	0.7	0.9	-	-	-	-	-	-
Germacrene D	1480	1478	0.1	0.4	-	-	-	-	-	-
Valencene	1494	1496	0.4	0.4	-	-	-	-	-	-
ε-Cadinene	1493	1497	t	0.2	-	-	-	-	-	-
α-Muurolene	1496	1499	t	0.4	-	-	-	-	-	-
γ-Cadinene	1507	1508	0.6	0.6	-	-	-	0.1	t	t
*cis*-Calamene	1517	1512	-	-	-	-	-	t	t	t
δ-Cadinene	1520	1516	0.7	0.4	-	-	-	0.1	t	t
Elemicin	1522	1524	3.3	4.0	11.7	23.2	24.2	14.0	12.7	15.6
(*E*)-Nerolidol	1553	1550	-	-	-	-	-	0.1	0.1	0.1
(*Z*)-Isoelemicin	1559	1564	-	t	0.1	0.2	0.1	0.1	0.1	0.2
Spathylenol	1572	1567	-	0.2	-	-	-	0.8	0.7	0.7
Caryophyllene oxide	1578	1573	t	t	-	-	-	-	-	-
(*E*)-Isoelemicin	1614	1621	-	2.8	3.4	0.8	0.4	13.0	15.8	12.5
T-Muurolol	1633	1630	-	t	0.1	0.1	0.1	0.2	0.2	0.2
α-Cadinol	1643	1642	-	t	0.2	0.2	0.1	0.4	0.3	0.3
Phenylpropanoids			22.3	24.9	76.5	69.5	69.7	69.2	71.0	60.6
MTH *			60.4	43.6	6.1	5.6	6.6	11.2	8.5	12.1
MTO			8.3	12.9	15.4	23.1	21.3	16.3	17.1	23.1
STH			5.5	11.1	t	t	t	0.3	t	t
STO			t	0.2	0.3	0.3	0.2	1.4	1.2	1.2
O			3.0	2.2	0.7	1.0	1.0	1.3	1.0	1.2

* MTH monoterpene hydrocarbons, MTO oxygenated monoterpenes, STH sesquiterpene hydrocarbons, STO oxygenated sesquiterpenes, O—other compounds, t—trace 0.05%, RI lit—retention index according to literature, RI exp—retention index experimentally estimated, FH—fresh herb, DH—dried herb, F10-E—fresh mater extract in 10% concentration, F20-E—fresh mater extract in 20% concentration, F30-E—fresh mater extract in 30% concentration, D2-E—dry mater extract in 2% concentration, D5-E—dry mater extract in 5% concentration, D10-E—dry mater extract in 10% concentration.

**Table 2:** Radical scavenging activity (RSC), EC_50_ values and total content of polyphenols (TCP) in *Artemisia dracunculus* extracts (absorbance—means ± SD).

Extract	Antioxidant Activity					TCP		
	Absorbance	*p*-Value	TEAC, mM	RSC %	EC_50_ %	Absorbance	*p*-Value	GAE mg/g
F10-E	1.085 ± 0.002 ^f^*	1.82 × 10^−29^	1.673	17.490	25.616	0.222 ± 0.024 ^a^	0.00002	0.528
F20-E	0.873 ± 0.001 ^d^		3.357	33.612		0.340 ± 0.016 ^d^		0.809
F30-E	0.504 ± 0.001 ^c^		6.291	61.673		0.342 ± 0.013 ^e^		0.814
D2-E	0.893 ± 0.001 ^e^		3.198	32.091	3.523	0.303 ± 0.028 ^b^		0.721
D5-E	0.364 ± 0.001 ^b^		7.402	72.319		0.339 ± 0.013 ^c^		0.807
D10-E	0.158 ± 0.001 ^a^		9.045	87.985		0.378 ± 0.008 ^f^		0.899

* Means ± SD marked with the same letters in columns do not differ significantly according to Tukey’s test at *p* < 0.05. For explanation of extracts used see Table 1.

**Table 3:** The number of aphids per one predator (larva of Syrphidae or Coccinellidae, adult of Coccinellidae) in the 2022–2024 seasons. Mean ± SE.

Treat- ment	Syrphidae Larva			Coccinellidae Larva			Coccinellidae Adult		
	2022	2023	2024	2022	2023	2024	2022	2023	2024
Ch	-	344 ± 92 ^a^*	320 ± 187 ^a^	120 ± 40 ^b^	105 ± 34 ^b^	271 ± 80 ^a^	106 ± 37 ^a^	384 ± 324 ^a^	1392 ± 1139 ^a^
Control	3980 ± 1250 ^b^	2024 ± 1187 ^a^	1870 ± 680 ^b^	174 ± 11 ^c^	335 ± 21 ^c^	1961 ± 766 ^a^	715 ± 69 ^c^	574 ± 274 ^a^	4942 ± 1827 ^a^
D2	2420 ± 504 ^ab^	310 ± 93 ^a^	528 ± 178 ^a^	72 ± 18 ^ab^	45 ± 8 ^ab^	1872 ± 793 ^a^	609 ± 252 ^b^	182 ± 56 ^a^	2162 ± 893 ^a^
D5	3606 ± 533 ^b^	594 ± 172 ^a^	535 ± 120 ^a^	51 ± 8 ^a^	29 ± 9 ^a^	2673 ± 1724 ^a^	115 ± 17 ^a^	241 ± 102 ^a^	2761 ± 1416 ^a^
D10	1052 ± 42 ^a^	384 ± 72 ^a^	321 ± 81 ^a^	32 ± 8 ^a^	34 ± 4 ^a^	517 ± 76 ^a^	111 ± 34 ^a^	126 ± 36 ^a^	1357 ± 520 ^a^
F10	1158 ± 184 ^a^	409 ± 160 ^a^	263 ± 63 ^a^	36 ± 1 ^a^	75 ± 31 ^ab^	3427 ± 2856 ^a^	222 ± 10 ^a^	164 ± 48 ^a^	1253 ± 530 ^a^
F20	923 ± 349 ^a^	375 ± 70 ^a^	445 ± 199 ^a^	59 ± 13 ^a^	78 ± 28 ^ab^	1002 ± 631 ^a^	191 ± 78 ^a^	215 ± 73 ^a^	1300 ± 286 ^a^
F30	2264 ± 193 ^ab^	343 ± 109 ^a^	525 ± 151 ^a^	59 ± 8 ^a^	52 ± 10 ^ab^	3105 ± 2617 ^a^	271 ± 69 ^a^	206 ± 49 ^a^	5909 ± 2800 ^a^

* Means ± SE marked with the same letters in columns do not differ significantly according to LSD test at *p* < 0.05. Treatments: Ch—broad beans subjected to standard chemical pest control practice; Control—broad beans non-protected, distilled water used in the same amount as in the case of extract treatments; D2—broad beans protected with 2% concentration of dry mater extract; D5—broad beans protected with 5% concentration of dry mater extract; D10—broad beans protected with 10% concentration of dry mater extract; F10—broad beans protected with 10% concentration of fresh mater extract; F20—broad beans protected with 20% concentration of fresh mater extract; F30—broad beans protected with 30% concentration of fresh mater extract.

**Table 4:** Pearson’s correlation coefficient between aphids and specific groups of predators.

Treatment	Syrphidae	Coccinellidae	Other Predators
Ch	0.279 *	0.159	−0.138
Control	0.464 *	0.302 *	0.118
D2	0.507 *	−0.018	0.049
D5	0.344 *	0.214 *	0.257 *
D10	0.483 *	−0.019	0.180
F10	0.544 *	0.109	0.210 *
F20	0.379 *	0.153	−0.071
F30	0.518 *	0.148	0.141
Without division	0.476 *	0.195 *	0.112 *

* significant at *p* < 0.05. For treatments, see Table 3.

## Data Availability

Data are contained within the article and Supplementary Materials.

## Supplementary Materials

The following supporting information can be downloaded at: https://www.mdpi.com/article/10.3390/molecules31193399/s1, Table S1: Anova results of the effect of treatments (T), year (Y) and their interaction (T*Y) as well as the effect of treatments in each specific year on the abundance of *Aphis fabae*, Syrphidae, Coccinellidae and other predators in broad bean; Table S2: Main effects of treatments (T), year (Y) and their interaction (T*Y) on the abundance of *Aphis fabae*, Syrphidae, Coccinellidae and other predators in broad bean; Table S3: Anova results of the effect of treatments (T), date (D) and their interaction (T*D) as well as the effect of treatments in each specific date of the observation on the abundance of *Aphis fabae*, Syrphidae, Coccinellidae and other predators in broad bean; Table S4: Anova results of the effect of treatments (T), year (Y) and their interaction (T*Y) as well as the effect of treatments in each specific year on the number of aphids per one predator (larva of Syrphidae or Coccinellidae, adult of Coccinellidae) in each specific year in broad bean; Table S5: Anova results of the effect of treatments (T), year (Y) and their interaction (T*Y) as well as the effect of treatments in each specific year on the yield of broad bean seeds in each specific year; Table S6: Main effects of treatments (T), year (Y) and their interaction (T*Y) on the yield of broad bean seeds.
