# Supplementary material for: Effect of Artemisia dracunculus L. Water Extracts on Aphis fabae Scop. and Its Predators in Field Conditions
Source: Molecules. 2026 Sep 24;31(19):3399. doi: 10.3390/molecules31193399 (PMC13665973; doi:10.3390/molecules31193399)
Supplement: Supplementary file 1 [file molecules-31-03399-s001.zip › molecules-4516818-supplementary.pdf]

Table S1. Anova results of the effect of treatments (T), year (Y) and their interaction (T*Y) as well as the effect of treatments in each specific year on the abundance of *Aphis fabae*, Syrphidae, Coccinellidae and other predators in broad bean.

Variables		*Aphis fabae*		Syrphidae		Coccinellidae		Other predators	
	df	F	p	F	p	F	p	F	p
T	7	17.991	0.000	6.339	0.000	6.193	0.000	3.172	0.003
Y	2	9.414	0.000	88.853	0.000	76.279	0.000	25.057	0.000
T*Y	14	1.810	0.033	1.756	0.041	2.733	0.001	2.851	0.000
				2022					
T	7	11.189	0.000	4.997	0.000	4.392	0.000	1.685	0.112
				2023					
T	7	3.852	0.001	3.010	0.005	1.017	0.420	0.921	0.491
				2024					
T	7	5.044	0.000	3.957	0.000	2.297	0.027	5.492	0.000

Table S2. Main effects of treatments (T), year (Y) and their interaction (T*Y) on the abundance of *Aphis fabae*, Syrphidae, Coccinellidae and other predators in broad bean.

Variables			*Aphis fabae*		Syrphidae		Coccinellidae		Other predators	
					(log (x+1) transformed)					
T	Ch		0.8896	a*	0.0146	a	0.0320	a	0.0088	a
T	Control		2.1441	e	0.0876	c	0.2097	c	0.0187	abc
T	D2		1.4660	cd	0.0858	c	0.1534	bc	0.0171	ab
T	D5		1.3844	bcd	0.0588	b	0.1475	b	0.0361	d
T	D10		1.1519	b	0.0616	b	0.1047	b	0.0267	bcd
T	F10		1.2665	bc	0.0697	bc	0.1209	b	0.0192	abc
T	F20		1.4208	cd	0.0720	bc	0.1458	b	0.0344	d
T	F30		1.5105	d	0.0783	bc	0.1538	bc	0.0302	cd
Y	2022		1.4061	b	0.0417	b	0.2579	c	0.0285	b
Y	2023		1.2117	a	0.0236	a	0.1157	b	0.0051	a
Y	2024		1.5311	b	0.1139	c	0.0458	a	0.0328	b
T*Y	Ch	2022	0.6856	a	0.0048	a	0.0496	abcd	0.0235	abcdefg
T*Y	Ch	2023	0.9852	abc	0.0055	a	0.0497	abcd	0.0013	a
T*Y	Ch	2024	0.9890	abc	0.0286	ab	0.0061	a	0.0022	ab
T*Y	Control	2022	2.6046	i	0.0926	cd	0.4698	i	0.0246	cdefg
T*Y	Control	2023	1.7619	gh	0.0391	ab	0.1169	bcdef	0.0029	abc
T*Y	Control	2024	2.0304	h	0.1161	de	0.0635	abcd	0.0245	cdefg
T*Y	D2	2022	1.5597	fg	0.0653	bc	0.3048	h	0.0304	defgh
T*Y	D2	2023	1.1604	bcdef	0.0275	ab	0.1436	def	0.0047	abc
T*Y	D2	2024	1.5948	g	0.1411	e	0.0387	abc	0.0147	abcdef
T*Y	D5	2022	1.3696	bcdefg	0.0262	ab	0.2894	gh	0.0221	abcdefg
T*Y	D5	2023	1.1791	bcdef	0.0172	a	0.1371	cdef	0.0055	abc
T*Y	D5	2024	1.5331	fg	0.1127	de	0.0408	abc	0.0677	i
T*Y	D10	2022	1.0126	abc	0.0348	ab	0.1903	efg	0.0375	gh
T*Y	D10	2023	0.9890	abc	0.0124	a	0.1064	abcde	0.0020	ab
T*Y	D10	2024	1.3719	cdefg	0.1158	de	0.0351	ab	0.0344	fgh
T*Y	F10	2022	1.1228	bcde	0.0307	ab	0.2186	efgh	0.0139	abcdef
T*Y	F10	2023	1.0900	abcd	0.0200	a	0.1124	bcdef	0.0065	abc
T*Y	F10	2024	1.4991	defg	0.1341	e	0.0485	abcd	0.0320	efgh
T*Y	F20	2022	1.5096	defg	0.0347	ab	0.2767	gh	0.0518	hi
T*Y	F20	2023	1.1819	bcdef	0.0297	ab	0.1212	bcdef	0.0098	abcde
T*Y	F20	2024	1.5090	defg	0.1301	de	0.0576	abcd	0.0369	gh
T*Y	F30	2022	1.3841	cdefg	0.0443	ab	0.2638	gh	0.0240	bcdefg

T*Y	F30	2023	1.3459	bcdefg	0.0376	ab	0.1379	cdef	0.0082	abcd
T*Y	F30	2024	1.7213	gh	0.1327	e	0.0764	abcd	0.0499	hi

* Means marked with the same letters for treatments, year and treatments x year, respectively do not differ significantly according to LSD test at $p < 0.05$. For treatments see Figure 3.

Table S3. Anova results of the effect of treatments (T), date (D) and their interaction (T*D) as well as the effect of treatments in each specific date of the observation on the abundance of *Aphis fabae*, Syrphidae, Coccinellidae and other predators in broad bean.

Date	Variables		***Aphis fabae***		**Syrphidae**		**Coccinellidae**		**Other predators**	
		df	F	p	F	p	F	p	F	p
					**2022**					
	T	7	16.211	0.000	7.161	0.000	7.474	0.000	2.022	0.054
Total	D	11	12.975	0.000	10.915	0.000	19.246	0.000	8.375	0.000
	T*D	77	0.921	0.655	1.158	0.211	0.944	0.607	0.674	0.976
11-May	T	7	3.470	0.019	1.000	0.466	0.859	0.557	0.438	0.864
15-May	T	7	6.887	0.001	2.274	0.082	0.836	0.573	0.758	0.629
18-May	T	7	5.040	0.004	0.436	0.865	1.674	0.186	1.629	0.198
23-May	T	7	6.878	0.001	3.229	0.025	1.568	0.215	0.667	0.697
25-May	T	7	1.150	0.382	2.178	0.094	0.639	0.718	0.691	0.679
29-May	T	7	2.049	0.111	4.017	0.010	1.061	0.430	0.668	0.696
01-June	T	7	0.915	0.520	0.534	0.796	1.223	0.346	0.588	0.756
05-June	T	7	1.430	0.261	1.097	0.411	1.794	0.158	0.605	0.744
15-June	T	7	5.474	0.002	0.738	0.644	1.708	0.177	0.635	0.721
19-June	T	7	2.676	0.049	2.452	0.065	2.216	0.089	0.651	0.709
25-June	T	7	0.773	0.619	0.859	0.557	1.170	0.372	2.115	0.102
					**2023**					
	T	7	13.520	0.000	3.919	0.001	2.293	0.030	0.879	0.525
Total	D	9	65.869	0.000	8.791	0.000	35.769	0.000	1.436	0.177
	T*D	63	0.976	0.534	0.998	0.491	0.655	0.972	0.768	0.884
13-May	T	7	4.140	0.009	1.000	0.466	1.694	0.181	0.555	0.781
17-May	T	7	4.014	0.010	1.598	0.207	0.939	0.504	1.000	0.466
20-May	T	7	3.473	0.019	0.772	0.619	1.251	0.333	0.715	0.661
24-May	T	7	3.361	0.021	0.690	0.679	0.578	0.764	0.869	0.551
27-May	T	7	11.408	0.000	0.626	0.728	0.683	0.685	1.000	0.466
31-May	T	7	2.933	0.035	1.571	0.215	2.820	0.041	1.210	0.352
03-June	T	7	1.271	0.324	1.514	0.232	1.927	0.131	0.440	0.863
06-June	T	7	1.413	0.267	1.169	0.373	1.159	0.378	0.861	0.556
10-June	T	7	1.524	0.229	1.427	0.262	0.615	0.736	0.822	0.584
13-June	T	7	2.038	0.113	1.750	0.167	1.060	0.431	0.686	0.683
					**2024**					
	T	7	24.592	0.000	9.951	0.000	3.415	0.002	5.847	0.000
Total	D	14	93.699	0.000	39.439	0.000	15.626	0.000	6.084	0.000
	T*D	98	1.678	0.001	0.949	0.611	0.657	0.991	0.506	1.000
30-April	T	7	2.108	0.103	1.149	0.383	1.000	0.466	0.874	0.547
04-May	T	7	2.772	0.043	1.104	0.407	1.110	0.403	0.715	0.661
08-May	T	7	1.346	0.293	0.857	0.559	1.000	0.466	0.631	0.724
11-May	T	7	1.691	0.182	1.285	0.318	1.155	0.380	0.664	0.699
14-May	T	7	2.172	0.094	1.189	0.362	0.723	0.655	0.664	0.699
18-May	T	7	3.283	0.023	0.325	0.932	0.606	0.743	1.812	0.154
21-May	T	7	4.306	0.007	1.109	0.404	1.112	0.402	0.885	0.540
26-May	T	7	3.096	0.029	1.410	0.268	0.532	0.798	0.717	0.659
29-May	T	7	7.362	0.000	2.034	0.113	1.066	0.428	0.946	0.500

03-June	T	7	1.630	0.198	1.555	0.219	0.758	0.629	0.923	0.514
05-June	T	7	6.370	0.001	5.297	0.003	0.856	0.560	0.761	0.627
09-June	T	7	6.549	0.001	1.008	0.462	0.530	0.800	0.319	0.934
12-June	T	7	6.112	0.001	2.256	0.084	1.052	0.436	0.829	0.578
16-June	T	7	3.941	0.011	0.967	0.487	0.864	0.554	2.318	0.078
19-June	T	7	1.488	0.240	1.731	0.172	0.787	0.608	0.859	0.557

Table S4. Anova results of the effect of treatments (T), year (Y) and their interaction (T*Y) as well as the effect of treatments in each specific year on the number of aphids per one predator (larva of Syrphidae or Coccinellidae, adult of Coccinellidae) in each specific year in broad bean.

Variables		**Syrphidae larva**		**Coccinellidae larva**		**Coccinellidae adult**	
	df	F	p	F	p	F	p
T	7	7.945	0.000	0.484	0.841	2.054	0.068
Y	2	27.385	0.000	8.391	0.001	21.814	0.000
T*Y	14	1.381	0.213	0.465	0.940	1.531	0.137
			2022				
T	7	4.695	0.008	7.593	0.000	5.499	0.002
			2023				
T	7	1.559	0.227	23.068	0.000	0.852	0.563
			2024				
T	7	3.678	0.021	0.409	0.861	1.708	0.192

Table S5. Anova results of the effect of treatments (T), year (Y) and their interaction (T*Y) as well as the effect of treatments in each specific year on the yield of broad bean seeds in each specific year.

Variables		**Yield**	
	df	F	p
T	7	45.704	0.000
Y	2	61.541	0.000
T*Y	14	8.391	0.000
		2022	
T	7	16.084	0.000
		2023	
T	7	9.367	0.000
		2024	
T	7	34.204	0.000

Table S6. Main effects of treatments (T), year (Y) and their interaction (T*Y) on the yield of broad bean seeds

**Variables**			**Yield [g m^{-2}]**	
T	Ch		701.19	d*
T	Control		162.70	a
T	D2		521.26	c
T	D5		380.95	b
T	D10		677.06	d
T	F10		521.04	c
T	F20		486.14	c
T	F30		538.78	c
Y	2022		381.73	a
Y	2023		491.17	b
Y	2024		623.01	c
T*Y	Ch	2022	592.08	fghi
T*Y	Ch	2023	575.17	efghi

T*Y	Ch	2024	936.33	k
T*Y	Control	2022	154.39	ab
T*Y	Control	2023	214.05	abc
T*Y	Control	2024	119.67	a
T*Y	D2	2022	251.32	bc
T*Y	D2	2023	568.58	efghi
T*Y	D2	2024	743.88	j
T*Y	D5	2022	203.86	abc
T*Y	D5	2023	457.92	de
T*Y	D5	2024	481.07	defg
T*Y	D10	2022	622.93	hij
T*Y	D10	2023	490.91	defg
T*Y	D10	2024	917.33	k
T*Y	F10	2022	487.73	defg
T*Y	F10	2023	600.78	ghi
T*Y	F10	2024	474.63	def
T*Y	F20	2022	298.19	c
T*Y	F20	2023	540.67	defgh
T*Y	F20	2024	619.58	hi
T*Y	F30	2022	443.37	d
T*Y	F30	2023	481.33	defg
T*Y	F30	2024	691.63	ij

***** Means marked with the same letters for treatments, year and treatments x year, respectively do not differ significantly according to LSD test at $p < 0.05$. For treatments see Figure 3.
